# Regulation of Neural Circuit Development by Cadherin-11 Provides Implications for Autism

**DOI:** 10.1523/ENEURO.0066-21.2021

**Published:** 2021-07-03

**Authors:** Jeannine A. Frei, Robert F. Niescier, Morgan S. Bridi, Madel Durens, Jonathan E. Nestor, Michaela B. C. Kilander, Xiaobing Yuan, Derek M. Dykxhoorn, Michael W. Nestor, Shiyong Huang, Gene J. Blatt, Yu-Chih Lin

**Affiliations:** 1 Program in Neuroscience, Hussman Institute for Autism, Baltimore, MD 21201; 2 Key Laboratory of Brain Functional Genomics (Ministry of Education and Shanghai), Institute of Brain Functional Genomics, School of Life Science and the Collaborative Innovation Center for Brain Science, East China Normal University, Shanghai, 200062, People's Republic of China; 3 John P. Hussman Institute for Human Genomics, University of Miami Miller School of Medicine, Miami, FL 33136

**Keywords:** autism spectrum disorder, cadherin-11, cadherin-8, Cdh11 knock-out mice, neural circuit formation, neuroligin-1

## Abstract

Autism spectrum disorder (ASD) is a neurologic condition characterized by alterations in social interaction and communication, and restricted and/or repetitive behaviors. The classical Type II cadherins cadherin-8 (Cdh8, CDH8) and cadherin-11 (Cdh11, CDH11) have been implicated as autism risk gene candidates. To explore the role of cadherins in the etiology of autism, we investigated their expression patterns during mouse brain development and in autism-specific human tissue. In mice, expression of cadherin-8 and cadherin-11 was developmentally regulated and enriched in the cortex, hippocampus, and thalamus/striatum during the peak of dendrite formation and synaptogenesis. Both cadherins were expressed in synaptic compartments but only cadherin-8 associated with the excitatory synaptic marker neuroligin-1. Induced pluripotent stem cell (iPSC)-derived cortical neural precursor cells (NPCs) and cortical organoids generated from individuals with autism showed upregulated CDH8 expression levels, but downregulated CDH11. We used *Cdh11* knock-out (KO) mice of both sexes to analyze the function of cadherin-11, which could help explain phenotypes observed in autism. *Cdh11*^−/−^ hippocampal neurons exhibited increased dendritic complexity along with altered neuronal and synaptic activity. Similar to the expression profiles in human tissue, levels of cadherin-8 were significantly elevated in *Cdh11* KO brains. Additionally, excitatory synaptic markers neuroligin-1 and postsynaptic density (PSD)-95 were both increased. Together, these results strongly suggest that cadherin-11 is involved in regulating the development of neuronal circuitry and that alterations in the expression levels of cadherin-11 may contribute to the etiology of autism.

## Significance Statement

Autism is a neurodevelopmental condition with high genetic and phenotypic heterogeneity. Multiple genes have been implicated in autism, including the cadherin superfamily of adhesion molecules, cadherin-8 and cadherin-11. This study first characterizes the expression profiles of mouse cadherin-8 and cadherin-11 to understand their potential roles in the development of neurons. The study further describes that the loss of cadherin-11 in mice results in altered levels of several synaptic proteins, including postsynaptic density (PSD)-95, neuroligin-1, and cadherin-8, changing the morphology and activity of excitatory neurons. The levels of cadherin-8 and cadherin-11 in human induced pluripotent stem cell (iPSC)-derived cortical neural precursor cells and cortical organoids of autistic individuals are both altered, strengthening that these two cadherins may be involved in autism etiology.

## Introduction

Autism is a neurodevelopmental condition characterized by marked qualitative changes in social interaction and communication as well as restricted and repetitive behaviors (American Psychiatric Association, 2013). Current estimates indicate that one in 54 children in the United States is affected (Maenner et al., 2020). Autism is characterized by both phenotypic and genetic heterogeneity. This genetic complexity is illustrated by the fact that no single gene associated with the condition contributes to >1% of the autism cases (Huguet et al., 2013; Jeste and Geschwind, 2014). Nevertheless, many of the genes implicated in the condition encode for synaptic cell adhesion molecules, scaffolding proteins and cytoskeletal regulators (Betancur et al., 2009; Bourgeron, 2015; Lin et al., 2016). These autism risk genes converge into selective cellular pathways that appear to be commonly affected, including neurite outgrowth, dendritic spine stability, synaptogenesis, and synaptic function (Betancur et al., 2009; Hussman et al., 2011; Chen et al., 2014; Bourgeron, 2015; Lin et al., 2016; Joensuu et al., 2018).

One large group of synaptic cell adhesion molecules, the cadherin superfamily, has been widely associated with neurodevelopmental disorders, including autism (Redies et al., 2012; Lin et al., 2016). The cadherin family comprises more than one hundred members, which are further divided into subfamilies, including classical Type I and Type II cadherins, clustered and non-clustered protocadherins, and atypical FAT cadherins (Hulpiau and van Roy, 2009; Hirano and Takeichi, 2012). Multiple studies have identified cadherins across all subfamilies as candidate risk genes for autism (Marshall et al., 2008; Morrow et al., 2008; Depienne et al., 2009; Wang et al., 2009; Willemsen et al., 2010; Chapman et al., 2011; Hussman et al., 2011; Pagnamenta et al., 2011; Sanders et al., 2011; Camacho et al., 2012; Neale et al., 2012; O’Roak et al., 2012; Girirajan et al., 2013; van Harssel et al., 2013; Crepel et al., 2014; Cukier et al., 2014; Kenny et al., 2014). The most-well studied classical Type I cadherin, N-cadherin, is broadly expressed in the central nervous system and has been implicated in multiple processes during nervous system development (Takeichi and Abe, 2005; Arikkath and Reichardt, 2008; Hirano and Takeichi, 2012; Friedman et al., 2015a; Seong et al., 2015). In contrast to classical Type I cadherins, the expression profile of classical Type II cadherins is more restricted to specific brain circuits and subcellular compartments (Korematsu and Redies, 1997; Suzuki et al., 1997; Inoue et al., 1998; Wang et al., 2019). The differential expression patterns of classical Type II cadherins allow them to confer sophisticated synaptic specificity (Basu et al., 2015).

The aim of the present study was to investigate the involvement of classical Type II cadherins in autism and to discern the contribution of these cadherins to neuronal development and function. We focused our study on the classical Type II cadherins cadherin-8 (Cdh8, CDH8) and cadherin-11 (Cdh11, CDH11) as these specific cadherins have been identified as autism risk genes in a genome-wide association study and by whole exome sequencing (Hussman et al., 2011; Cukier et al., 2014). In addition, other studies have identified rare mutations and SNP variants in *CDH8* and *CDH11* genes, respectively, in individuals with autism (Pagnamenta et al., 2011; Crepel et al., 2014). Here, we first examined the expression profiles and binding partners of cadherin-8 and cadherin-11 in developing mouse brains. Our results indicated that although these two cadherins show similar spatial expression and interact with each other, they may engage in divergent cellular pathways. We further used *Cdh11* knock-out (KO) mice to identify potential phenotypes that may be altered in autism as, similarly, human induced pluripotent stem cell (hiPSC)-derived neural precursor cells (NPCs) and organoids generated from individuals with autism displayed differentially altered expression levels of cadherin-8 and cadherin-11. Together, the data suggest that depletion of cadherin-11 causes altered neural circuit development that may drive aspects of autism pathophysiology.

## Materials and Methods

### Animals

C57BL/6 mice were purchased from the animal facility of the University of Maryland School of Medicine Program in Comparative Medicine (Baltimore, MD). *Cdh11^tm1Mta^/*HensJ mice were purchased from The Jackson Laboratory (Horikawa et al., 1999). Mice were housed and cared for by the AAALAC accredited program of the University of Maryland School of Medicine. Female mice were group-housed and male mice were singly housed with *ad libitum* food and water accessibility under a standard 12/12 h light/dark cycle. Neonatal mice of both sexes were euthanized for the preparation of neuronal and non-neuronal cultures. To match the mixed-gender condition in cultures animals of both sexes were used for biochemistry. All experiments were reviewed and approved by the Institutional Care and Use Committees (IACUC) of the University of Maryland School of Medicine and the Hussman Institute for Autism, and were performed in accordance with the animal care guidelines of the National Institutes of Health.

### Antibodies

Primary and secondary antibodies used in this study are listed in Tables 1, 2, respectively. The specificity of the antibodies was carefully examined before conducting the experiments (Extended Data Fig. 1-1).

**Table 1:** Primary antibodies

Primary antibodies						
Antibody	Host, isotype	Immunogen	Source	Catalog number	Clone no./RRID	Dilution
Cadherin-8	Mouse	N terminus, mouse	DSHB	CAD8-1	RRID:AB_2078272	0.5 μg/ml (WB), 3–4 μg/ml (ICC), 2 μg (IP)
Cadherin-8	Goat, IgG	C terminus, human	Santa Cruz	sc-6461	C-18/RRID:AB_2078271	1:500
Cadherin-11	Mouse, IgG_1_	C terminus, human	Thermo Fisher Scientific	32-1700	5B2H5/RRID:AB_2533068	2 μg/ml (WB), 7 μg (IP)
PSD-95	Mouse, IgG_2a_	Amino acids 77–299, human	Neuro Mab	75-028	K28/43/RRID:AB_2307331	1:100,000 (WB)
PSD-95	Guinea pig, IgG	Amino acids 64–247, mouse	Synaptic Systems	124014	RRID:AB_2619800	1:500 (ICC)
Syntaxin-1	Mouse, IgG_2a_	N terminus, rat	Synaptic Systems	110111	78.3/RRID:AB_887848	1:10,000
Synapsin-1	Rabbit, IgG	Native protein purified from bovine brain	Abgent	P17599	N/A	1:1000
GAT-1	Rabbit, IgG	C terminus, rat	Phospho Solutions	880-GAT1	RRID:AB_2492119	1:200
Neuroligin-1	Mouse, IgG_1_	Amino acids 718–843, rat	Neuro Mab	75-160	N97A/31/RRID:AB_2235964	1:100
N-cadherin	Mouse, IgG_1_	Amino acids 802–819, mouse	BD Transduction Labs	610920	32/RRID:AB_2077527	1:1000
β-Catenin	Mouse, IgG_1_	Amino acids 571–781, mouse	BD Transduction Labs	610153	14/RRID:AB_397554	1:700
GAPDH	Rabbit, IgG	C terminus, human	Cell Signaling Technology	5174	D16H11/RRID:AB_10622025	1:1000
β-Actin	Mouse, IgG_2a_	N terminus	Millipore Sigma	A5441	AC-74/RRID:AB_476743	1:5000
HA	Rabbit, IgG	Amino acids 98–106 of human influenza hemagglutinin	Millipore Sigma	H6908	RRID:AB_260070	1:1000 (WB), 1:25 (ICC), 4 μg (IP)
C-myc	Mouse, IgG_1_	Amino acids 408–439 of human p62*^c^*^-^*^myc^*	Millipore Sigma	M4439	9E10/RRID:AB_291324	1:3000 (WB), 1:500 (ICC)
Flag	Mouse, IgG_2b_	DYKDDDDK tag	Thermo Fisher Scientific	MA191878	FG4R/RRID:AB_1957945	1:500 (ICC)

List of primary antibodies used for Western blotting (WB), immunocytochemistry (ICC), and immunoprecipitation (IP). PSD-95, postsynaptic density protein-95; GAT-1, sodium-dependent and chloride-dependent GABA transporter-1; GAPDH, glyceraldehyde 3-phosphate dehydrogenase; aa, amino acid.

**Table 2:** Secondary antibodies

Secondary antibodies						
Antibody	Host, isotype	Immunogen	Source	Catalog number	Clone no./RRID	Dilution
Anti-rabbit IgG, HRP conjugate	Goat, IgG	IgG rabbit	Cell Signaling Technology	7074P2	RRID:AB_2099233	1:7500
Anti-mouse IgG, HRP conjugate	Goat, IgG	IgG mouse	Thermo Fisher Scientific	A16072	RRID:AB_2534745	1:5000
Anti-goat IgG, HRP conjugate	Donkey, IgG	IgG goat	Thermo Fisher Scientific	A15999	RRID:AB_2534673	1:5000
Anti-mouse Alexa Fluor 488	Donkey, IgG	IgG (H + L) mouse	Thermo Fisher Scientific	A21202	RRID:AB_141607	1:1000
Anti-mouse Alexa Fluor 647	Donkey, IgG	IgG (H + L) mouse	Thermo Fisher Scientific	A31571	RRID:AB_162542	1:1000
Anti-rabbit Alexa Fluor 488	Donkey, IgG	IgG (H + L) rabbit	Thermo Fisher Scientific	A21206	RRID:AB_2535792	1:1000
Anti-rabbit Alexa Fluor 568	Donkey, IgG	IgG (H + L) rabbit	Thermo Fisher Scientific	A10042	RRID:AB_2534017	1:1000
Anti-rabbit Alexa Fluor 647	Donkey, IgG	IgG (H + L) rabbit	Thermo Fisher Scientific	A31573	RRID:AB_2536183	1:1000
Anti-guinea pig Alexa Fluor 488	Donkey, IgG	IgG (H + L) guinea pig	Jackson ImmunoResearch	706545148	RRID:AB_2340472	1:1000
Anti-guinea pig Alexa Fluor 568	Goat, IgG	IgG (H + L) guinea pig	Thermo Fisher Scientific	A11075	RRID:AB_2534119	1:1000

List of secondary antibodies used for Western blotting and immunocytochemistry. HRP, horseradish peroxidase.

### Plasmids

Myc-flag-tagged full-length *Cdh8* was purchased from Origene (plasmid #MR218916). *Cdh8* was expressed under the CMV promoter in the pCMV6 vector. Flag-tagged full-length *Cdh11* was expressed under the EF-1α promoter in the pBos vector (gift from Megan Williams, University of Utah). HA-tagged *Nlgn1* plasmid was a gift from Peter Scheiffele (Addgene plasmid #15260; RRID:Addgene_15260; Chih et al., 2006). *Nlgn1* was expressed under the chicken β-actin promoter in the pCAAGs vector. pLL3.7-GFP was a gift from Luk Parijs (Addgene plasmid #11795; RRID:Addgene_11795; Rubinson et al., 2003).

### Cell cultures and transfection

Non-neuronal cell cultures were prepared from postnatal day (P)0 C57BL/6 mouse cortices and cultured in DMEM growth medium (Invitrogen catalog #11960044) supplemented with 10% fetal bovine serum (FBS; Millipore Sigma catalog #F4135), 2 mm L-glutamine (Invitrogen catalog #25030081), and 1% penicillin/streptomycin (Invitrogen catalog #15140122). Primary neuronal cultures were prepared from P0 C57BL/6 mouse cortex (three to four animals per culture) or hippocampus (8–10 animals per culture). Hippocampal cultures from *Cdh11^tm1Mta^/*HensJ mice were prepared from individual pups at P0. The genotype of each pup was determined after the culture was prepared. Only *Cdh11* wild-type (WT) and KO cultures were used for experiments. In brief, brain tissue was dissected and meninges were removed. Tissue was digested in papain and cells were dissociated and plated on surfaces coated with 20 μg/ml poly-D-lysine (Millipore Sigma catalog #P6407). Cortical and hippocampal cultures were maintained in serum-free Neurobasal-A media (Invitrogen catalog #10888022) containing 2 mm L-glutamine (Invitrogen catalog #25030081), 1% penicillin/streptomycin (Invitrogen catalog #15140122), and 2% B27 supplement (Invitrogen catalog #17504044). For Western blot analysis, non-neuronal cells were harvested at 14 d *in vitro* (DIV) and cortical neurons were harvested at different time points: 1, 3, 7, and 14 DIV. *Cdh11* WT and KO cultures were harvested at 4, 7, and 14 DIV. Neuro-2A (N2a; mouse neuroblastoma cell line; ATCC) cells were maintained in DMEM growth medium (Invitrogen catalog #11960044) supplemented with 10% FBS (Millipore Sigma catalog #F4135), 2 mm L-glutamine (Invitrogen catalog #25030081), and 1% penicillin/streptomycin (Invitrogen catalog #15140122). N2a cells were transfected with full-length plasmids using Lipofectamine 3000 (Invitrogen catalog #L3000015) according to the manufacturer’s protocol. Cells were harvested 48 h post-transfection.

### Western blot analysis

At the indicated points in development, mice of both sexes were sacrificed and brains were quickly dissected. Either whole brains or different brain areas, including forebrain, cortex, hippocampus, cerebellum and thalamus/striatum were collected. Brain tissue was snap-frozen in liquid nitrogen. All tissues and cells were lysed in radioimmunoprecipitation assay (RIPA) buffer (Cell Signaling Technologies catalog #9806S) supplemented with PMSF (Cell Signaling Technologies catalog #8553S) and protease and phosphatase inhibitor cocktail (Thermo Fisher Scientific catalog #78 442). Protein concentration was determined using Pierce BCA protein assay kit (Thermo Fisher Scientific catalog #23227) and measured by the Tecan Spark 10 M multimode microplate reader. Ten micrograms of protein samples from brain or cell lysates were run on 10% Tris-glycine SDS-PAGE gels and transferred to a PVDF membrane. Membranes were blocked in 5% milk/TBS-T followed by incubation of primary antibodies overnight and secondary horseradish peroxidase (HRP)-coupled antibodies for 1 h at room temperature. Blots were imaged with the ChemiDoc Touch Imaging System (Bio-Rad) and band densitometries were analyzed using the Image Lab software (Bio-Rad). Intensities were normalized to either glyceraldehyde 3-phosphate dehydrogenase (GAPDH) or β-actin signals.

### Synaptic fractionation

Synaptic plasma membrane (SPM) and postsynaptic density (PSD) fractions were prepared according to Bermejo et al. (2014). In brief, brains of P21 mice were quickly removed and the forebrain was dissected on ice. Brain tissues were homogenized in 0.32 m sucrose in 4 mm HEPES (pH 7.4) at 900 rpm with 12 strokes using a glass-Teflon tissue homogenizer. Removal of the nuclear fraction (P1) was achieved by low-speed centrifugation at 900 × *g* for 10 min. The supernatant (S1) was collected and centrifuged at 10,000 × *g* for 15 min to yield crude synaptosomal fraction (P2) and cytosol/light membranes in the supernatant (S2). P2 pellet was lysed in ddH_2_O by hypo-osmotic shock and centrifuged at 25,000 × *g* for 20 min to obtain pelleted synaptosomes (P3) and vesicular fraction (S3). The vesicular fraction was pelleted by ultracentrifugation at 165,000 × *g* for 2 h. Synaptosomes (P3) were layered on top of a discontinuous sucrose gradient. Ultracentrifugation of the gradient at 150,000 × *g* for 2 h yielded the SPM fraction. SPM was collected and pelleted by ultracentrifugation at 200,000 × *g* for 30 min. To prepare PSD fraction, SPM was incubated in 0.5% Triton X-100 for 15 min followed by centrifugation at 32,000 × *g* for 30 min. All centrifugation steps were performed at 4°C. Fractions were analyzed by Western blotting and intensities of cadherin-8, cadherin-11, PSD-95, and syntaxin-1 positive signals in P3, SPM, PSD, and S3 fractions were normalized to the total protein input.

### Co-immunoprecipitation (IP)

P14 forebrain and transfected and untransfected N2a cells were homogenized in lysis buffer containing 20 mm Tris, pH 7.5, 150 mm NaCl and 1% Triton X-100 supplemented with PMSF (Cell Signaling Technologies catalog #8553S) and protease and phosphatase inhibitor cocktail (Thermo Fisher Scientific catalog #78442). Protein extract (0.5 mg, standardized to 1 mg/ml) was precleared with 50 μl Protein-G Sepharose four Fast Flow (Millipore Sigma catalog #GE17-0618-01) or rProtein A Sepharose Fast Flow (Millipore Sigma catalog #GE17-1279-01) for 1 h at 4°C. Precleared supernatant was incubated with 2 μg anti-cadherin-8 (DSHB catalog #CAD8-1), 7 μg anti-cadherin-11 (Thermo Fisher Scientific catalog #32-1700) or 4 μg anti-HA antibodies (Millipore Sigma catalog #H6908), and mouse or rabbit IgGs for 2 h at 4°C. Samples were precipitated with 50 μl of preequilibrated Protein-G Sepharose four Fast Flow or rProtein A Sepharose Fast Flow for 1 h at 4°C with gentle mixing. Immunoprecipitates were washed three times in lysis buffer and eluted by boiling in 50 μl sample buffer. Overexpressed myc-flag-tagged cadherin-8 was immunoprecipitated using the Pierce c-My-tag IP/Co-IP kit according to the manufacturer’s protocol (Thermo Fisher Scientific catalog #23620). Co-immunoprecipitated proteins were determined by Western blot analysis.

### Immunocytochemistry

Surface staining was performed on low-density hippocampal cultures (20,000 cells/2 cm^2^) at 15 DIV. Cells were washed with artificial CSF (aCSF) containing 124 mm NaCl, 5 mm KCl, 1.23 mm NaH_2_PO_4_, 26 mm NaHCO_3_, 10 mm Dextrose, 1 mm MgCl_2_, 1 mm CaCl_2_, and supplemented with 10% BSA. Cells were incubated with aCSF containing primary antibody for 1 h at 20°C. After washing with aCSF/10% BSA, cells were incubated with secondary antibody for 1 h at 20°C before fixation with 4% paraformaldehyde for 15 min (Noel et al., 1999; Gu and Huganir, 2016). For total staining, N2a and primary cells were fixed with 4% paraformaldehyde, permeabilized with 0.1% Triton X-100 and incubated with primary antibodies overnight and secondary antibodies for 1 h at room temperature. Cells were labeled with DAPI and coverslips were mounted on glass slides with ProLong Diamond antifade mounting solution (Thermo Fisher Scientific catalog #P36934) before imaging. Images were taken using a Zeiss LSM-780 scanning confocal microscope with a 40× objective/1.30 EC-plan-neofluar oil or a 63× objective/1.40 plan-apochromat oil for N2a cells, and a 63× objective/1.40 plan-apochromat oil and 4× zoom for primary cells. For co-localization analysis, cadherin-8-positive puncta were manually counted and classified as either co-localizing with PSD-95 or GAT-1 (partially or totally overlapping puncta), being adjacent to PSD-95 or GAT-1 (puncta that were in close proximity and touching each other) or cadherin-8 puncta that were PSD-95-negative or GAT-1-negative.

### Direct stochastic optical reconstruction microscopy (dSTORM) imaging

A total of 67,000 cells prepared from P0 C57BL/6 mouse hippocampus were plated onto Fluorodish 35-mm dishes with 23 mm #1.5-glass bottoms (catalog #FD35-100). At 15 DIV, cells were surface stained with either cadherin-8 or neuroligin-1 primary antibody and Alexa Fluor 647-conjugated secondary antibody as described above. After surface staining, cells were fixed, permeabilized and immunostained with PSD-95 primary antibody and Alexa Fluor 488-conjugated secondary antibody and maintained in PBS before imaging. Imaging was performed using a Nikon N-STORM microscope, using the following buffer system: 150 mm tris-HCl (pH 8.0), 100 mm MEA-HCl (Millipore Sigma catalog #M6500), 3% Oxyfluor (Millipore Sigma catalog #SAE0059), and 2% DL-lactate (Millipore Sigma catalog #L1375; Nahidiazar et al., 2016). A minimum of 20,000 frames were obtained using 100% laser power of 488- and 647-nm channels with 16-ms exposure time using elliptical lens for 3D analysis. STORM processing was performed using the Fiji (NIH) plug-in ThunderSTORM (Ovesný et al., 2014). For nearest neighbor analysis, the ThunderSTORM CBC function was used to compare the cadherin-8 or neuroligin-1 localization tables to the corresponding PSD-95 table. Individual molecules of either cadherin-8 or neuroligin-1 were manually identified, and the closest molecule of PSD-95 was quantified; 50 fluorescence particles per image were examined, unless fewer than that were available for analysis. For a negative control, numbers between 0 and 400 were randomly generated using Microsoft Excel. For comparison between random, cadherin-8, and neuroligin-1, values were binned at 10-nm intervals and plotted using GraphPad Prism 8 software (GraphPad Prism Software, RRID: SCR_002798).

### Morphometric analysis

Cultured *Cdh11* WT and KO hippocampal neurons (300,000 cells/2 cm^2^) were transfected at 3 DIV or 9 DIV with the pLL3.7-GFP vector (Addgene catalog #11795) using Lipofectamine 2000 (Invitrogen catalog #11668-019) and fixed at 4, 7, or 15 DIV with 4% paraformaldehyde, respectively. Z-stack images were taken using Zeiss LSM-780 scanning confocal microscope with a 63× objective/1.40 plan-apochromat oil at 1500 × 1500 resolution and stitched with 2 × 2 frames. For dendritic spine analysis of 15 DIV cultures, two to three dendrite segments per neuron were randomly selected from primary, secondary and tertiary branches on the apical dendrite. The number of dendritic spines (≤2 μm in length) was quantified using Fiji (NIH). To quantify spine size, high resolution confocal images were taken with an image pixel size of 64 nm. Only secondary dendrites were analyzed. Spine size was measured using Fiji (NIH), by drawing an ROI around the entire spine, starting at the base of the spine at the dendritic shaft. For analysis of dendritic morphology, neurons were traced and the total dendrite length (TDL) and branch tip number were quantified using Fiji (NIH) with NeuronJ plugin (Meijering et al., 2004); 15 DIV neurons were further analyzed using Fiji (NIH) with Sholl analysis plugin (Ferreira et al., 2014). From the center of the cell body, concentric circles having 10-μm increments in radius were defined and the number of traced dendrites crossing each circle was quantified. The complexity of dendritic arbors was analyzed by the area under the curve (AUC) using GraphPad Prism 8 software (GraphPad Prism Software, RRID: SCR_002798).

### Calcium imaging

*Cdh11* WT and KO mouse hippocampal neurons were seeded in triplicates at a density of 30,000 cells per well on a 96-well plate coated with 20 μg/ml poly-D-lysine (Millipore Sigma catalog #P6407). At 7 DIV, neurons were infected with the IncuCyte NeuroBurst Orange lentivirus under a synapsin promoter (Essen Bioscience Sartorius catalog # 4736). After 24 h, virus was removed by changing the media. Cells were imaged at 15 DIV for 24 h using the IncuCyte S3 system (Essen Bioscience Sartorius catalog #4763). Using the IncuCyte S3 2019A software (Essen Bioscience Sartorius) the following parameters were calculated: number of active neurons, mean correlation of activity, mean burst strength, and mean burst rate. The total number of active neurons was identified by the analysis definition. For the mean correlation of activity, the temporal pattern of the change in fluorescent intensity for each active neuron was compared with every other active neuron in the image. A value between −1 and 1 was generated, with 0 being completely random (no correlation) and 1 being identical patterns of change in fluorescent intensity (highly correlated). Fisher r-to-z transformation was applied to assess the significance between correlation coefficients. The mean burst strength was analyzed by integrating the AUC divided by its duration. This value was calculated for each burst individually and then averaged for each active neuron, followed by averaging across the entire image. To calculate the mean burst rate the total number of bursts for each active neuron was divided by minutes of scan time, followed by averaging the values for all active neurons across the entire image. The total number of cells was obtained by counting DAPI-positive nuclei. IncuCyte NeuroLight Orange lentivirus (Essen Bioscience Sartorius catalog #4758) was used to measure the infection rate of *Cdh11* WT and KO hippocampal neurons. Neurons were infected at 7 DIV, and the virus was removed after 24 h by changing the media. Neurons were fixed with 4% paraformaldehyde at 16 DIV and imaged using Evos Auto 2.0 system (Invitrogen) with a 10× objective. The number of infected cells was counted and subsequently normalized to the total cell number determined by DAPI.

### Electrophysiology

Electrophysiological recordings were conducted using *ex vivo* brain slices of *Cdh11* WT and KO littermate mice between P7 and P9 or P21 and P24. Mice were anesthetized with isoflurane, decapitated, and brains removed into ice-cold sucrose cutting solution containing the following: 215 mm sucrose, 2.5 mm KCl, 1.25 mm NaH_2_PO_4_, 2.8 mm NaHCO_3_, 7 mm dextrose, 3 mm Na-Pyruvate, 1 mm Na-ascorbate, 0.5 mm CaCl_2_, and 7 mm MgCl_2_ (pH 7.4, bubbled with 95% CO_2_/5% O_2_). Near-horizontal slices containing hippocampus, 300 μm thick, were sectioned with a vibrating microtome (VT1200S, Leica Biosystems). After cutting, slices were transferred into a warmed recovery chamber filled with bubbled aCSF containing the following: 125 mm NaCl, 2.5 mm KCl, 1.25 mm NaH_2_PO_4_, 25 mm NaHCO_3_, 25 mm dextrose, 2 mm CaCl_2_, 1 mm MgCl_2_, 3 mm Na-pyruvate, and 1 mm Na-ascorbate. After recovering for 30 min at a temperature of 34°C, the slices recovered for at least another 30 min at room temperature before recording. For recording, slices were transferred to a recording chamber superfused with the same aCSF used in the recovery chamber, maintained at 32°C. Miniature EPSCs (mEPSCs) were recorded in voltage-clamp mode (V_h_ = −76 mV) in the presence of 100 μm DL-AP5, 10 μm tetrodotoxin, and 20 μm gabazine (channel blockers acquired from Tocris). Glass pipettes pulled to a resistance of 2–6 MΩ were filled with internal solution containing the following: 130 mm K-gluconate, 10 mm KCl, 10 mm HEPES, 0.2 mm EGTA, 4 mm MgATP, 0.5 mm Na_2_GTP, 10 mm Na_2_-phosphocreatine, and 5 mm QX-314 chloride, pH adjusted to 7.3 with KOH, 280–290 mOsm. Pyramidal neurons in stratum pyramidal of CA1 in dorsal hippocampal slices were visually identified for recording. Only cells with a series resistance ≤30 MΩ, input resistance >80 MΩ, and a resting membrane potential ≤ −50 mV were accepted for final analysis. Whole-cell parameters were monitored throughout the recording with a 100-ms, −10-mV step delivered every 30 s. Recordings were made using an Axon MultiClamp 700B amplifier (Molecular Devices). Data were filtered at 2 kHz and digitized at 10 kHz with a National Instruments digital-analog converter under the control of Igor Pro software (WaveMetrics, RRID: SCR_000325). The frequency, amplitude, rise and decay times, and charge of mEPSCs were analyzed with Mini Analysis software (Synaptosoft, RRID: SCR_002184), with a threshold of 3× RMS noise for event discrimination. In P21–P24 mice, 100–200 well-isolated events were used for these analyses in WT neurons; because of extremely low event frequency, a minimum of 50 events were used in KO neurons. In P7–P9 mice, because of extremely low event frequency in CA1, all events within a 5-min window of recording for each cell were selected for analysis.

### Ascertainment and clinical criteria of autistic and control individuals

All of the autism spectrum disorder (ASD) patients were recruited through the University of Miami John P. Hussman Institute for Human Genomics with informed consent obtained from all participants under a University of Miami Miller School of Medicine Institutional Review Board approved protocol and all experiments were performed in compliance with the guidelines and regulations of the institutional biosafety committee. ASD individuals contributing samples for the generation of iPSCs (110, 134, 691, 700, 709, 710, 725, 732) were ascertained following an ASD diagnosis. The core inclusion criteria were as follows: (1) individuals were between 3 and 21 years of age; (2) individuals had a presumptive clinical diagnosis of ASD; (3) an expert clinical determination of an ASD diagnosis was determined using DSM-V (American Psychiatric Association, 2013) criteria supported by the Autism Diagnostic Interview-Revised (ADI-R; Lord et al., 1994); and (4) individuals had an intelligence quotient (IQ) equivalent >35 or developmental level >18 months as determined by the Vineland Adaptive Behavior Scale (VABS; Sparrow, Sparrow et al., 2005). All diagnoses were based on review by a panel consisting of experienced clinical psychologists and a pediatric medical geneticist. As appropriate, IQ measures were obtained for the individuals following administration of any of several measures [e.g., age-appropriate Wechsler scale, Leiter intelligence test, or Mullen Scales of Early Learning (MSEL)] or derived from medical records. All of the ASD individuals were non-syndromic and sporadic with polygenetic background as shown by whole exome sequencing (Cukier et al., 2014). The ASD lines, 110, 134, 691, 709, 725, 732, had been derived and validated in our previous publication (DeRosa et al., 2018). Briefly, the iPSC lines were derived from peripheral blood mononuclear cells (PBMCs) isolated from the whole blood by density centrifugation. The cells were reprogrammed using the CytoTune iPS 2.0 Sendai Reprogramming kit (Thermo Fisher Scientific catalog #A16517) according to the manufacturer’s protocol. Individual clonal iPSC lines from each participant were validated for pluripotency by immunocytochemistry for pluripotency markers (OCT3/4, NANOG, SOX2, TRA-1-81) and genomic stability confirmed by G band karyotype (WiCell) as previously published (DeRosa et al., 2018). Patients 700 and 710 were ascertained using the same approach as the other individuals and the iPSC lines derived and validated in the same manner as the previously published samples (DeRosa et al., 2018). Control samples were obtained following informed consent under an IRB approved protocol (University of Miami) from cognitively normal individuals that were between 18 and 30 years of age. These individuals had no history of ASD or other neurologic disorders (e.g., schizophrenia, major depressive disorder). The control samples were slightly older than the individuals with ASD to ensure that they did not have a diagnosis of autism or a related neurodevelopmental disorder, such as schizophrenia that is often diagnosed during adolescence. PBMCs were isolated from whole blood obtained from the study participant. The reprogramming for the control lines was performed in the same manner as that used for the ASD samples. These samples were screened for pluripotency and genomic stability in the same way as the ASD samples according to our published protocol (DeRosa et al., 2018). All the samples, both the ASD and the control samples, were from non-Hispanic white (NHW) males.

### hiPSC-derived cortical NPC cultures

iPSC lines were derived from peripheral blood obtained from eight individuals with autism and from four typically developing controls (Table 3; DeRosa et al., 2018). PBMCs were isolated and cultured in suspension until transduction using Oct4, Sox2, Klf4, and c-Myc Cytotune Sendai viruses (Thermo Fisher Scientific catalog #A16517; DeRosa et al., 2012). After PBMC transduction into iPSCs the media was supplemented with 10 μm CHIR99021 (Stemgent catalog #04000410), 1 μm PD325901 (Stemgent catalog #040006), 1 μm thiazovivin (Stemgent catalog #040017), and 10 μm Y27632 (Stemgent catalog #04001210) for 7 d. At day 7, the media with small molecules was transitioned to mTeSR1 full stem cell maintenance media (Stemcell Technologies catalog #85850) with the media being changed daily. iPSC colonies were plated onto mouse embryonic feeders (MEFs) and grown for 7 d. Colonies were selected showing proliferating cell clusters, indicative of reprogrammed cells. To derive cortical progenitor neurons, selected iPSC colonies were dissociated via a 7-min treatment with Accutase in the presence of 20 μm Y27632 and MEF feeders were removed using 0.1% gelatin as previously described (Nestor et al., 2015; Phillips et al., 2017). Dissociated iPSCs were -exposed to media containing small molecules, e.g., 10 μm Y27632, 10 μm SB431542 (Stemgent catalog #04001010), 1 μm dorsomorphin (Stemgent catalog #040024), and 1 μm thiazovivin, within growth media as described previously (DeRosa et al., 2012). After patterning and neural induction, iPSC-derived neuron progenitors were expanded using six-well plates coated with 15 μg/ml poly-L-ornithine (Millipore Sigma catalog #P4957) and 10 μg/ml laminin (Invitrogen catalog # 230171015) within an enriched medium containing 1:1 mixture of DMEM/F12 (with L-glutamine; Thermo Fisher Scientific catalog #11320033) and Neurobasal medium (minus phenol red; Invitrogen catalog #12348017), 5 μm forskolin (Millipore Sigma catalog #F6886), 60 ng/ml progesterone (Millipore Sigma catalog #P8783), 16 μg/ml putrescine (Millipore Sigma catalog #P7505), 5 μg/ml *N*-acetyl-L-cysteine (Millipore Sigma catalog #A8199), 1% insulin-transferrin-selenium-A (Invitrogen catalog #41400045), 1% B-27 supplement (Invitrogen catalog #12587010), 0.5% N2 supplement (Invitrogen catalog #17502048), 1% penicillin/streptomycin (Thermo Fisher Scientific catalog #15140122), 30 ng/ml tri-iodothyronine (Millipore Sigma catalog #T6397), 40 ng/ml thyroxine (Millipore Sigma catalog #T1775), 0.5% non-essential amino acids, 100 μg/ml bovine-serum albumin (Millipore Sigma catalog #A4161), and 0.5% GlutaMAX (Invitrogen catalog #35050061). Cells were harvested at 19 DIV in RIPA buffer (Cell Signaling Technologies catalog #9806S) supplemented with PMSF (Cell Signaling Technologies catalog #8553S) and protease and phosphatase inhibitor cocktail (Thermo Fisher Scientific catalog #78442), and triplicates of each control and autism line were analyzed by Western blotting.

**Table 3:** Case information of control and autism-derived iPSC lines

	Sample ID	Gender	Diagnosis	Genes
Autism	110	M	Autism	VPS13B, EFCAB5, TRIM55
	700	M	Autism	RBFOX1
	725	M	Autism	CEP290, NINL, SOS2, TRIM55, ZMYND17, BTN2A2, MDC1, FBXO40, KIAA1949, SLC8A3, TSPYL5
	732	M	Autism	CLCN2, F13A1, JARID2, STXBP5, C12orf73, C20orf118, FGD6
	134	M	Autism	CPZ, PRICKLE1, TOPOR5
	691	M	Autism	COL6A3, SLIT3, C2orf85, AB13BP, UIMC1
	709	M	Autism (twin)	RBFOX1
	710	M	Autism (twin)	RBFOX1
Control	574	M	-	-
	321	M	-	-
	322	M	-	-
	324	M	-	-

Sample identity number, gender, diagnosis, and affected genes are listed for each iPSC line analyzed. Data was obtained from the University of Miami John P. Hussman Institute for Human Genomics (Hussman et al., 2011; Cukier et al., 2014).

### Organoid culture and mRNA quantitation via quantitative PCR (qPCR)

Cortical organoid cultures were generated from four control and six autism lines (Table 3) as previously described in Durens et al. (2020). In brief, PBMC-derived iPSCs were grown on MEF feeder layers and neural induction was performed as described above. At 14 DIV, aggregates were transferred to 0.4 μm PTFE inserts (Millipore catalog #PICM0RG50) and cultured in enriched medium containing DMEM/F12 medium (Invitrogen catalog #11320033) and Neurobasal medium (1:1; Invitrogen catalog #12348017), 2% B27 supplement (Invitrogen catalog #12587010), 0.5% N2 supplement (Invitrogen catalog #17502048), 0.5% non-essential amino acids (Invitrogen catalog #11140050), 1% SATO mix (progesterone (Millipore Sigma catalog #P8783), putrescine (Millipore Sigma catalog #P7505), tri-idothyronine (Millipore Sigma catalog #T6397), thyroxine (Millipore Sigma catalog #T1775), and bovine serum albumin (Millipore Sigma catalog #A4161), *N*-acetyl-L-cysteine (Millipore Sigma catalog #A8199), 1% insulin-transferrin-selenium-A (Thermo-Fisher catalog #51300044), 1% penicillin/streptomycin (Thermo Fisher Scientific catalog #15140122), and supplemented with the following neurotrophic factors: 20 ng/ml BDNF (Peprotech catalog #45002), 20 ng/ml NT3 (Peprotech catalog #45003), 20 ng/ml β-NGF (Peprotech catalog #45001), 20 ng/ml FGF (StemGent catalog #030002), 1 μg/ml laminin (ThermoFisher catalog #23017015), 5 μm forskolin (Millipore Sigma catalog #F6886), and 2 μg/ml heparin (Millipore Sigma catalog #H3149). At 30 DIV, 2 μm DAPT (Stemgent catalog #040041) was added, and FGF, β-NGF, and heparin were removed to induce terminal differentiation. At 45 DIV, DAPT was removed. Organoids were cultured for a total of 60 d, and then transferred to RNAprotect Cell Reagent (QIAGEN catalog #76 526) for RNA stabilization. Two organoids were pooled per biological replicate and triplicates of each control and autism line were analyzed. Samples were stored at −80°C until processed. Samples in RNAprotect were thawed on ice and centrifuged at 5000 × *g* for 5 min after which supernatant was removed. Total RNA was extracted using the RiboPure kit (Thermo Fisher Scientific catalog #AM1924) following manufacturer’s specifications. RNA concentration was determined using NanoDrop 8000 Microvolume UV-Vis Spectrophotometer (Thermo Fisher Scientific). DNA-*free* DNA removal kit (Thermo Fisher Scientific catalog #AM1906) was used to remove residual DNA. Reverse transcription (RT) was performed using 250 ng of DNase-treated RNA using the iScript cDNA synthesis kit (Bio-Rad catalog #1708890) and the resulting RT reactions were diluted 1:5 with nuclease-free water. qPCR was performed for the following genes: CDH8 (forward, 5′-ACA GCG AAT TTT GAA CCG CTC-3′; reverse, 5′-TCC TCC CGG TCA AGT CTT TTT-3′) and CDH11 (forward, 5′-AGA GGT CCA ATG TGG GAA CG-3′, reverse: 5′-GGT TGT CCT TCG AGG ATA CTG-3′). GAPDH was used as a reference gene (Genecopoeia catalog #HQP006940). qPCR was performed with 2× All-in-One qPCR Mix (Genecopoeia catalog #QP001) using the following reaction mix: 10 μl All-in-One qPCR Mix (2×), 2 μl of 20 μm primer, 5 μl nuclease-free water, and 5 μl of cDNA at 1:5 dilution. Reactions were incubated at 95° for 10 min, followed by 40 cycles of 95° for 10 s, 60° for 20 s, and 72° for 30 s using the CFX96 Real-time System (Bio-Rad). Melt curves were generated after amplification by increasing temperature from 72° to 95° at 0.5° increments. All reactions were performed in duplicates. Relative quantities for each gene were analyzed using the comparative Ct method. Fold changes were calculated relative to the average Ct values for control samples.

### Experimental design and statistical analysis

**Figure 1.**
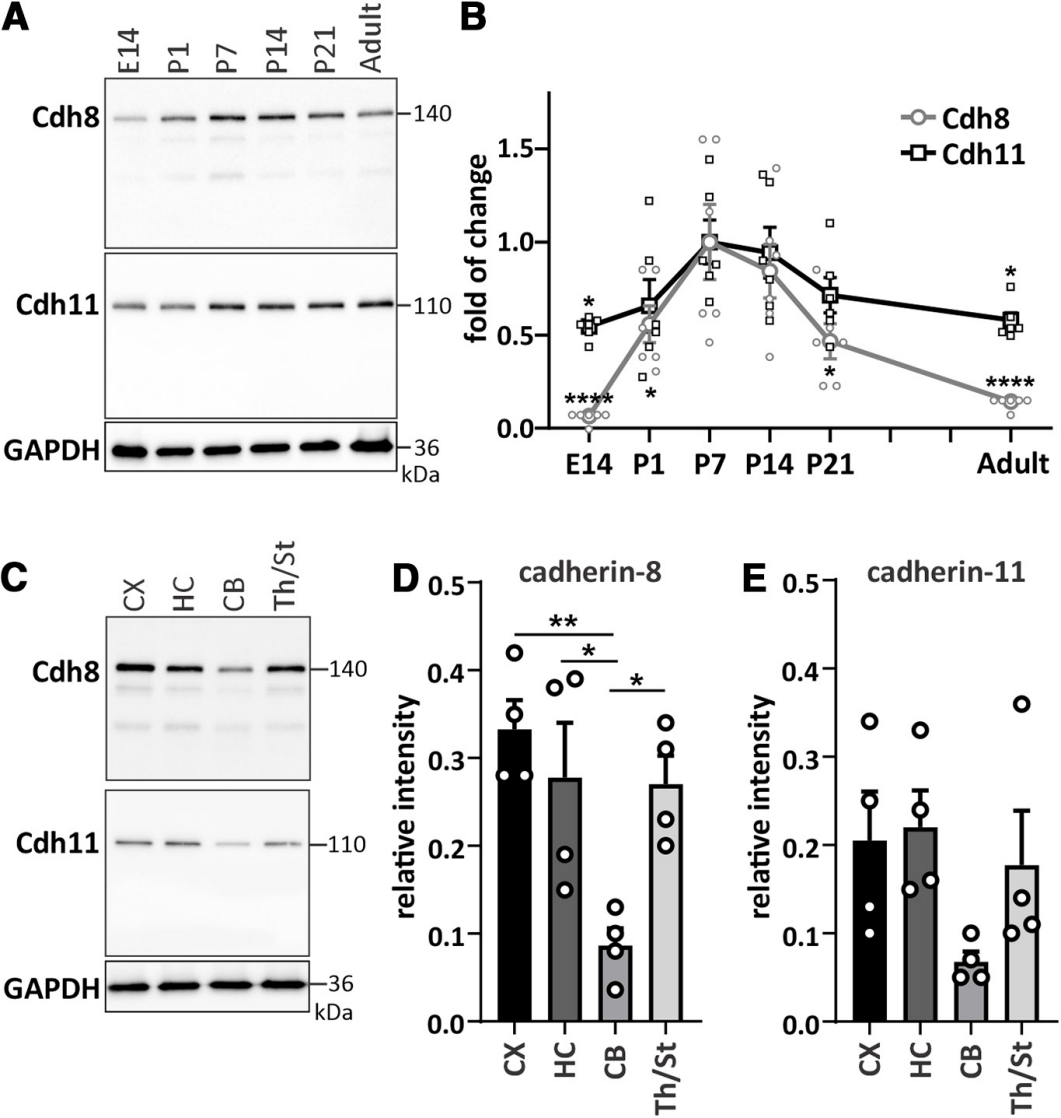
Cadherin-8 and cadherin-11 show similar temporal and spatial expression patterns. ***A***, Temporal expression profile of cadherin-8 and cadherin-11 in mouse whole brain harvested at different developmental ages. Adult mice were five months old. ***B***, Line graph of temporal expression of the two cadherins. Values were normalized to P7. Cdh8: *p =* *0.046 (P1), *p =* *0.0123 (P21), ****p <* *0.0001; Cdh11: *p =* *0.017 (E14), *p =* *0.0296 (adult), one-way ANOVA with Dunnett’s multiple comparisons test. *N* = 6 whole brains per age from three independent litters. ***C***, Expression profiles of cadherin-8 and cadherin-11 in different brain areas, including cortex (CX), hippocampus (HC), cerebellum (CB), and thalamus/striatum (Th/St) at P14. Quantification of (***D***) cadherin-8 and (***E***) cadherin-11 expression in different brain regions; *p =* *0.0259 between HC and CB, *p =* *0.0326 between CB and Th/St, **p =* *0.0048 between CX and CB, one-way ANOVA with Tukey’s multiple comparisons test. *N* = 4 samples per brain area with two to three pooled brain areas per sample. Cadherin signals were normalized to GAPDH. The specificity of anti-cadherin-8 and anti-cadherin-11 antibodies were tested in control experiments (see Extended Data Figure 1-1).

All data are reported as mean ± SEM as either line graphs or bar graphs with scatter plots, or as box and whisker plots displaying all data points from minimum to maximum. For Western blot analysis P7 time point was used as the baseline for normalization in Figure 1*B*; for comparison of protein expression in different synaptic fractions, values were normalized to total protein input; for comparison of protein expression in *Cdh11* KO and WT samples, values were normalized to WT. Statistical analysis was performed using GraphPad Prism 8 software (GraphPad Prism Software, RRID: SCR_002798). Unpaired two-tailed *t* test was performed when comparing two groups and one-way ANOVA with Tukey’s or Dunnett’s multiple comparisons test was used to compare differences between three to six groups; *p* values were considered significant if ≤0.05. All *p* values, t values, *F* values, degrees of freedom (DF), sample sizes (*N*) as well as statistical tests performed are reported in Table 4 and in the figure legends. Sample sizes were predetermined before each experiment. Normality tests were run to confirm normal distribution of the samples.

**Table 4:** Statistical results

Figure	Test	*F*_(DFn,DFd)_	*t* value	*p* value	DF
1*B*, Cdh8	One-way ANOVA	*F*_(5,30)_ = 10.26		<0.0001	5
1*B*, Cdh11	One-way ANOVA	*F*_(5,30)_ = 3.384		0.0153	5
1*D*	One-way ANOVA	*F*_(3,12)_ = 7.040		0.0055	3
1*E*	One-way ANOVA	*F*_(3,12)_ = 2.176		0.1438	3
2*A*	One-way ANOVA	*F*_(3,8)_ = 6.339		0.0165	3
2*B*	One-way ANOVA	*F*_(3,8)_ = 2.104		0.1781	3
2*C*	*t* test		5.332	0.0031	5
2*D*	*t* test		4.534	0.0062	5
3*B*, PSD-95	One-way ANOVA	*F*_(4,15)_ = 32.22		<0.0001	4
3*B*, Cdh8	One-way ANOVA	*F*_(4,15)_ = 1.747		0.1921	4
3*B*, Cdh11	One-way ANOVA	*F*_(4,15)_ = 34.99		<0.0001	4
3*B*, syntaxin-1	One-way ANOVA	*F*_(4,15)_ = 13.81		<0.0001	4
4*C*	One-way ANOVA	*F*_(2,114)_ = 10.10		<0.0001	2
4*D*	One-way ANOVA	*F*_(2,90)_ = 78.09		<0.0001	2
4*J*	One-way ANOVA	*F*_(2,57)_ = 36.49		<0.0001	2
5*A*	*t* test		3.950	0.0027	10
5*B*	*t* test		2.485	0.0323	10
5*C*	*t* test		2.342	0.0473	8
5*D*	*t* test		6.748	0.0001	8
6*B*, primary	*t* test		1.128	0.2638	59
6*B*, secondary	*t* test		1.346	0.1833	59
6*B*, tertiary	*t* test		0.9198	0.3614	59
6*B*, total	*t* test		0.4928	0.6239	59
6*C*	*t* test		0.5255	0.6014	54
6*E*	*t* test		2.286	0.0269	46
6*F*, TDL	*t* test		2.207	0.0323	46
6*F*, tip number	*t* test		2.387	0.0212	46
6*G*, TDL	*t* test		1.745	0.0845	88
6*G*, tip number	*t* test		0.5062	0.6140	88
6*H*, TDL	*t* test		0.2949	0.7689	68
6*H*, tip number	*t* test		0.8764	0.3839	68
6*J*	*t* test		10.06	<0.0001	12
6*K*	*t* test		2.945	0.0123	12
6*L*	*t* test		4.004	0.0017	12
6*M*	*t* test		3.496	0.0044	12
6*N*	*t* test		0.9961	0.3389	12
6*O*, PSD-95	*t* test		0.2046	0.8420	10
6*O*, Nlgn1	*t* test		0.05644	0.9561	10
6*O*, Cdh8	*t* test		1.582	0.1447	10
6*O*, N-cadherin	*t* test		0.3710	0.7184	10
6*P*, PSD-95	*t* test		1.944	0.0805	10
6*P*, Nlgn1	*t* test		0.7755	0.4560	10
6*P*, Cdh8	*t* test		1.674	0.1252	10
6*P*, N-cadherin	*t* test		0.9067	0.3859	10
7*B*	*t* test		3.481	0.0022	21
7*C*	*t* test		3.427	0.0027	20
7*D*	*t* test		2.541	0.0259	12
7*E*	*t* test		0.8066	0.4290	21
7*G*	*t* test		2.254	0.0456	11
7*H*	*t* test		2.234	0.0472	11
7*l*	*t* test		3.796	0.0030	11
7*J*	*t* test		1.289	0.2238	11
8*B*	*t* test		0.1577	0.8775	11
8*C*	*t* test		0.4992	0.6260	13
8*D*	*t* test		2.591	0.0160	24
8*E*	*t* test		3.101	0.0049	24
8*F*	*t* test		3.947	0.0006	24
8*G*	*t* test		0.7550	0.4576	24
9*C*	*t* test		0.5769	0.5688	27
9*D*	*t* test		0.3030	0.7642	27
9*E*	*t* test		0.4087	0.6860	27
9*F*	*t* test		0.5385	0.5946	27
9*G*	*t* test		0.2913	0.7731	27
9*J*	*t* test		4.776	<0.0001	46
9*K*	*t* test		2.486	0.0166	46
9*L*	*t* test		1.984	0.0532	46
9*M*	*t* test		0.0004	0.9997	46
9*N*	*t* test		0.5073	0.6144	46

Detailed information about statistical test, *F* values, *t* values, *p* values, and degree of freedom (DF) for all experiments performed in this study.

10.1523/ENEURO.0066-21.2021.f1-1 Extended Data Figure 1-1 Specificity of antibodies. ***A***, Specificity of antibodies was determined by Western blotting (WB). WB of untransfected N2a cells, N2a cells transfected (TF) with myc-flag-tagged *Cdh8* or flag-tagged *Cdh11* and P14 mouse brain tissues were probed with mouse anti-Cdh8, goat anti-Cdh8, or mouse anti-Cdh11 antibodies. Each antibody recognizes prominent bands of predicted molecular weight [Cdh8: 140 kDa (precursor), 90 kDa (mature); Cdh11: 110 kDa], each band representing either the overexpressed protein in N2a cells or the endogenous protein in brain tissues. Note that Cdh8 and Cdh11 are not endogenously expressed in N2a cells. ***B***, Cross-reactivity of antibodies. WB of N2a cells transfected with myc-flag-tagged *Cdh8* or flag-tagged *Cdh11* were probed with the same antibodies as in ***A***. Each antibody specifically recognizes the predicted cadherin and is not cross-reacting with the other cadherin examined. GAPDH served as loading control. ***A***, ***D***, Specificity of antibodies tested by immunofluorescence. myc-flag-tagged *Cdh8-*transfected (***C***) or HA-tagged *Nlgn-1*-transfected (***D***) N2a cells were fixed and stained with the mouse anti-Cdh8 or mouse anti-Nlgn-1 antibody. These antibodies specifically recognize the overexpressed cadherin-8 (cyan) and its flag-tag (magenta) and the overexpressed neuroligin-1 (cyan) and its HA-tag (magenta), respectively, with characteristic localization to cell membranes and cell-cell contacts. No signals were detectable in untransfected N2a cells that were incubated with primary and secondary antibodies. DAPI signal is depicted in blue. Scale bars: 10 μm (***C***) and 20 μm (***D***). Download Figure 1-1, TIF file.

### Data availability statement

The dataset supporting the conclusions of this study are available on reasonable request by the corresponding authors.

## Results

### Cadherin-8 and cadherin-11 show similar temporal and spatial expression patterns

We first compared the overall protein levels of classical Type II cadherins, cadherin-8 and cadherin-11, in C57BL/6 WT mouse whole brain samples taken at different ages of development from embryonic day (E)14 to P21, as well as in adulthood, by Western blotting (Fig. 1*A*). The anti-cadherin-8 and anti-cadherin-11 antibodies were validated for specificity by Western blotting (Extended Data Fig. 1-1*A*,*B*). Cadherin-8 and cadherin-11 both exhibited relatively low expression at E14 but their levels increased postnatally (Fig. 1*B*). Cadherin-8 levels readily increased at P1 and reached more than a 10-fold increase by P7 and P14. The expression of Cadherin-8 dropped after P14 and remained at low levels in adulthood. Cadherin-11 levels reached an ∼2-fold increase by P7 and P14 compared with E14, but were reduced to P1 level by P21 and to E14 level by adulthood (Fig. 1*B*). Comparison to the developmental timeline showed that cadherin-8 and cadherin-11 expression correlated to time points during which processes of dendrite development and synaptogenesis are prevalent, suggesting that they may play a role in these processes. We then analyzed the protein levels in specific brain regions at P14 (Fig. 1*C*). Cadherin-8 expression was significantly higher in the cortex, hippocampus and thalamus/striatum compared with the cerebellum (Fig. 1*D*). Cadherin-11 showed a similar expression pattern, although differences between regions were not statistically significant (Fig. 1*E*).

**Figure 2.**
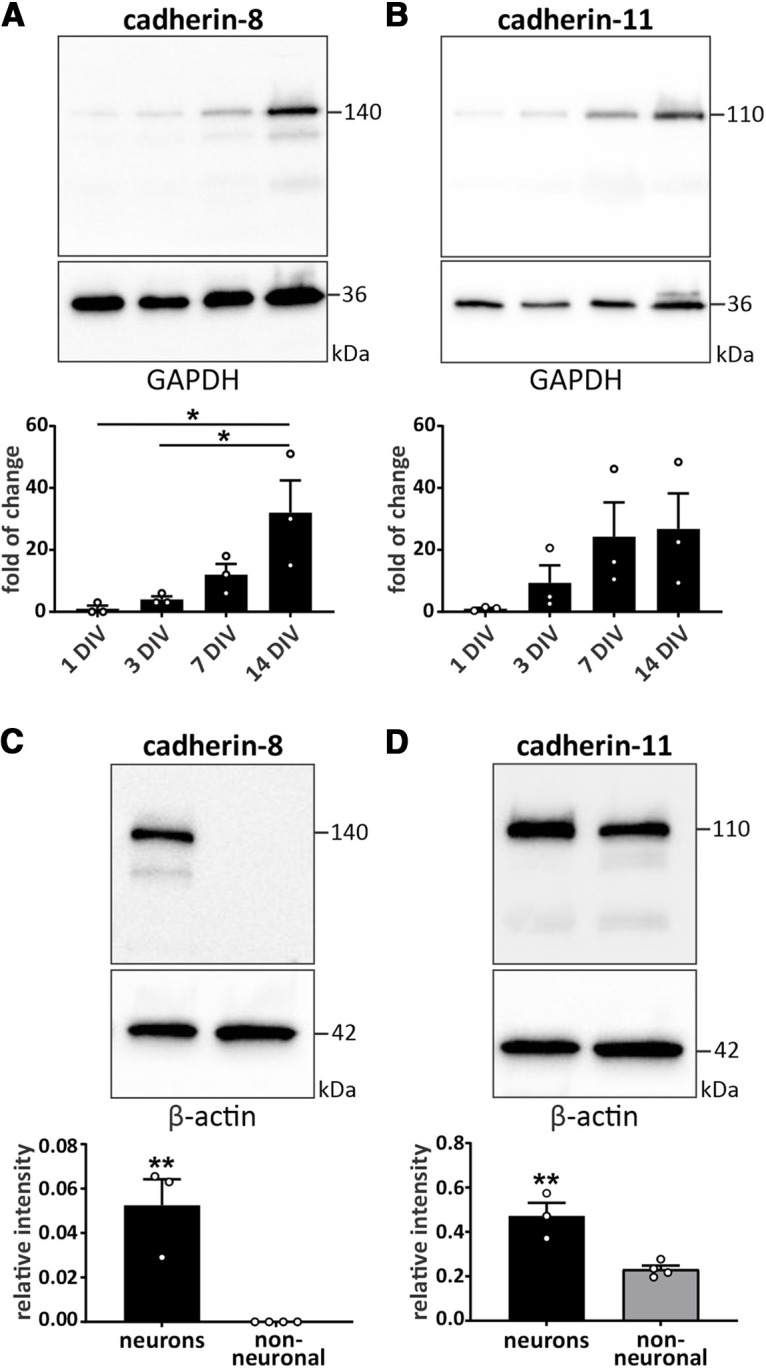
Cadherin-8 and cadherin-11 are preferentially expressed in neurons. Temporal expression pattern of (***A***) cadherin-8 and (***B***) cadherin-11 in cortical neurons harvested at 1, 3, 7, and 14 DIV; *p =* *0.0177 between 1 DIV and 14 DIV, *p =* *0.0299 between 3 DIV and 14 DIV, one-way ANOVA with Tukey’s multiple comparisons test. *N* = 3 independent cultures. Relative expression levels of (***C***) cadherin-8 and (***D***) cadherin-11 in cultured neurons and non-neuronal cells; **p <* *0.01, unpaired two-tailed *t* test. *N* = 3 neuronal and 4 non-neuronal cell cultures. Cells were cultured for 14 DIV before harvest. Cadherin signals were normalized to GAPDH (***A***, ***B***) and β-actin (***C***, ***D***).

To further interrogate which brain cell type(s) express cadherins, protein levels of cadherin-8 and cadherin-11 were measured in primary cortical neurons and non-neuronal cells cultured from mouse cortices. The temporal expression patterns of these two cadherins in primary cortical neurons showed a gradual and significant increase of cadherin-8 and a trend toward increased expression of cadherin-11 from 1 to 14 DIV (Fig. 2*A*,*B*). In contrast to neurons, cadherin-8 was virtually undetectable in primary non-neuronal cells (Fig. 2*C*). Cadherin-11 was expressed in both cortical neurons and non-neuronal cells, but the protein levels in neurons were 2-fold greater than in non-neuronal cells (Fig. 2*D*). These results demonstrate that both cadherins are preferentially expressed in neurons.

### Cadherin-8 and cadherin-11 localize to synaptic compartments but reveal distinct interaction profiles

**Figure 3.**
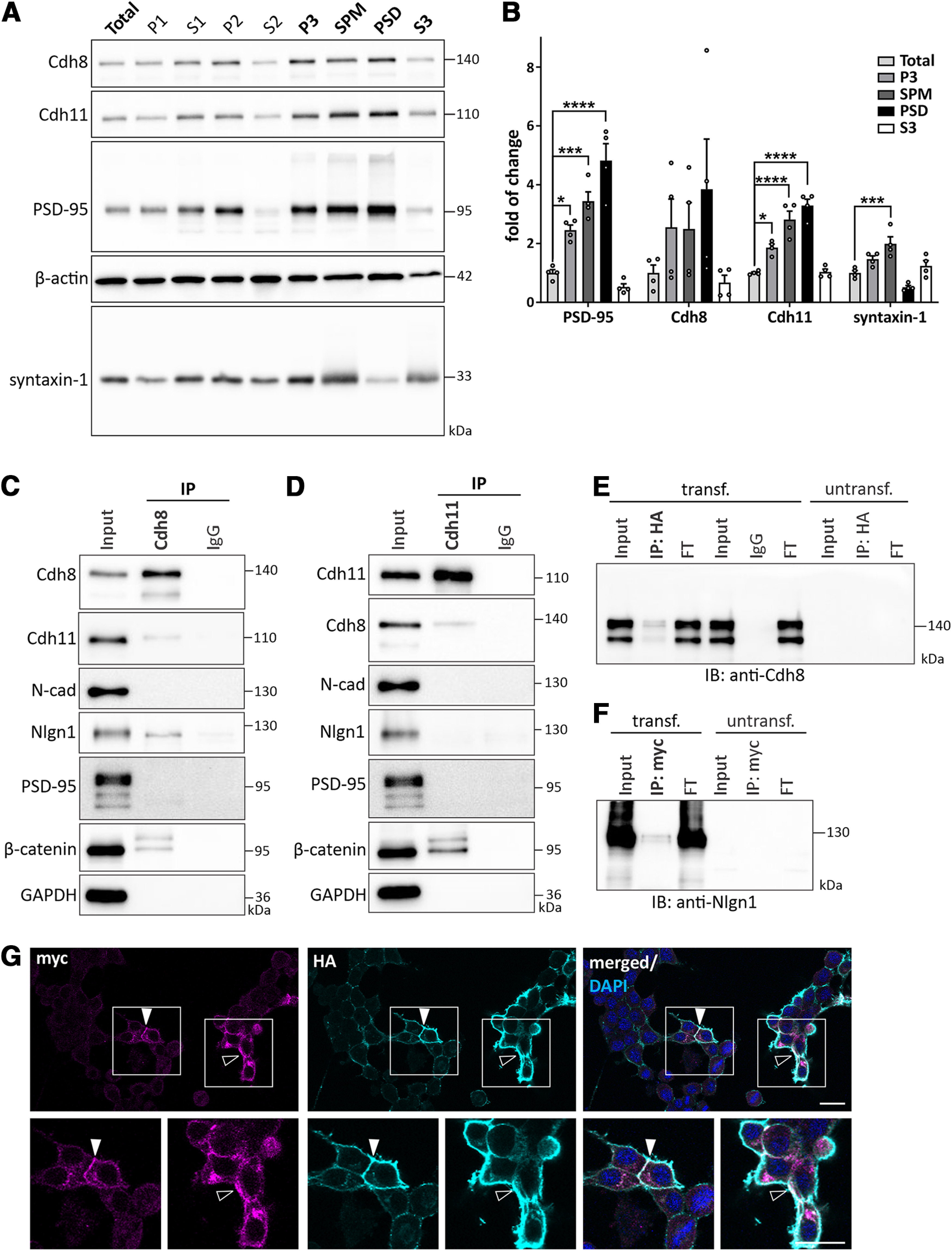
Cadherin-8 and cadherin-11 are expressed in synaptic compartments and cadherin-8 interacts with neuroligin-1. ***A***, Forebrain tissues were subjected to synaptic fractionation analysis to determine the subcellular localization of cadherin-8 and cadherin-11. Markers (PSD-95 and syntaxin-1) were probed as control for purity of the fractionation. Total: total protein input, P1: nuclear, S1: cytosol/membranes, P2: crude synaptosome, S2: cytosol/light membranes, P3: synaptosome, SPM: synaptic plasma membrane, PSD: postsynaptic density, S3: synaptic vesicles. ***B***, Quantification of protein enrichment in P3, SPM, PSD, and S3 fractions compared with total protein input. PSD-95: *p =* *0.0159, ***p =* *0.0002, ****p <* *0.0001; Cdh8: *p *=* *0.6618 (total vs SPM), *p *=* *0.1673 (total vs PSD); Cdh11: *p =* *0.0113, ****p <* *0.0001; Syntaxin-1: ***p =* *0.0009; one-way ANOVA with Dunnett’s multiple comparisons test. *N* = 8 P21 mice, two pooled forebrains per sample. Representative Western blotting of IP of (***C***) cadherin-8 and (***D***) cadherin-11 from P14 forebrain tissues. Cadherin-8 binds to cadherin-11, β-catenin, and neuroligin-1, whereas cadherin-11 binds to cadherin-8 and β-catenin, but not neuroligin-1. *N* = 3 independent co-IPs. Representative Western blotting of (***E***) IP of neuroligin-1-HA immunoblotted for Cdh8 and (***F***) IP of cadherin-8-myc immunoblotted for Nlgn1 from N2a cells overexpressing cadherin-8-myc and neuroligin-1-HA. Cadherin-8-myc is present in the IP of neuroligin-1-HA and neuroligin-1-HA is present in the IP of cadherin-8-myc. Cadherin-8 and neuroligin-1 are not expressed endogenously in N2a cells. FT, flow-through. *N* = 3 independent co-IPs. ***G***, Immunocytochemistry of N2a cells overexpressing cadherin-8-myc (magenta) and neuroligin-1-HA (cyan), counterstained for DAPI (blue). Cadherin-8-myc and neuroligin-1-HA show partial co-localization at cell-cell contacts (arrowheads) and at the cell membrane (open arrowheads). Boxed areas are magnified. Scale bar: 20 μm.

We next performed subcellular fractionation of forebrain tissue to isolate SPM and PSD to determine whether cadherins are enriched in synaptic compartments (Fig. 3*A*,*B*; Table 5). The distribution of PSD-95, syntaxin-1, and β-actin were evaluated to confirm successful separation and purity of different subcellular fractions. PSD-95 was used as a positive control for protein enrichment in SPM and PSD fractions, it was significantly enriched in SPM and PSD fractions compared with total protein input (Fig. 3*B*; Table 5). Syntaxin-1 was significantly enriched in SPM and, consistent with its presynaptic expression and function in vesicle release, expression in synaptic vesicle fraction (S3) was higher compared with PSD fraction (Fig. 3*B*; Table 5). Cadherin-8 showed a trend of enrichment in SPM and PSD, although the difference was not significant (Fig. 3*B*; Table 5). Cadherin-11 showed significant enrichment in both, SPM and PSD fractions (Fig. 3*B*; Table 5). Together, these results show that both cadherin-8 and cadherin-11 are present in presynaptic as well as postsynaptic membranes and localize to PSDs.

**Table 5:** Enrichment of protein expression in synaptic fractions

	P3	SPM	PSD	S3
PSD-95	2.45 (±0.17)	3.44 (±0.32)	4.82 (±0.57)	0.52 (±0.11)
Cdh8	2.55 (±0.96)	2.49 (±0.90)	3.85 (±01.70)	0.67 (±0.25)
Cdh11	1.86 (±0.12)	2.82 (±0.29)	3.29 (±0.22)	1.03 (±0.09)
Syntaxin-1	1.47 (±0.11)	1.99 (±0.24)	0.51 (±0.06)	1.24 (±0.18)

Mean values (±SEM) of enrichment of PSD-95, cadherin-8, cadherin-11, and syntaxin-1 in different subcellular fractions, including synaptosome (P3), synaptic plasma membrane (SPM), postsynaptic density (PSD), and synaptic vesicle fraction (S3).

Since cadherin-8 and cadherin-11 were both expressed in the PSD fraction, we performed co-IP experiments using anti-Cdh8 (Fig. 3*C*) and anti-Cdh11 antibodies (Fig. 3*D*) to determine the association of these two cadherins with specific postsynaptic proteins. Both, cadherin-8 and cadherin-11, immunoprecipitated β-catenin, the conserved intracellular binding partner of classical cadherins (Seong et al., 2015). Also, both cadherins interacted with each other, consistent with previous reports showing cadherin-8-cadherin-11 interactions in cultured cells and through biophysical analysis (Shimoyama et al., 2000; Brasch et al., 2018). Neither cadherin-8 nor cadherin-11 co-immunoprecipitated with PSD-95 or N-cadherin. Previous studies found cooperative functions of classical cadherins with the excitatory synaptic adhesion molecule neuroligin-1 (Nlgn1) during neural circuit development (Stan et al., 2010; Aiga et al., 2011; Yamagata et al., 2018). This prompted us to investigate whether cadherin-8 and cadherin-11 physically interact with neuroligin-1. Interestingly, neuroligin-1 was detected in the immunoprecipitate of cadherin-8 (Fig. 3*C*), but not cadherin-11 (Fig. 3*D*). We confirmed this interaction *in vitro* by co-expressing myc-tagged *Cdh8* together with HA-tagged *Nlgn1* in N2a cells that do not endogenously express these proteins (Fig. 3*E*,*F*; Extended Data Fig. 1-1*C*,*D*). Using either anti-HA or anti-myc antibodies to selectively pull down neuroligin-1 and cadherin-8, respectively, we found that neuroligin-1 co-immunoprecipitated cadherin-8 and vice versa. Consistent with these findings, immunostaining of N2a cells overexpressing myc-tagged *Cdh8* and HA-tagged *Nlgn1* showed a partial co-localization of these two proteins at the plasma membrane and at cell-cell contacts (Fig. 3*G*).

**Figure 4.**
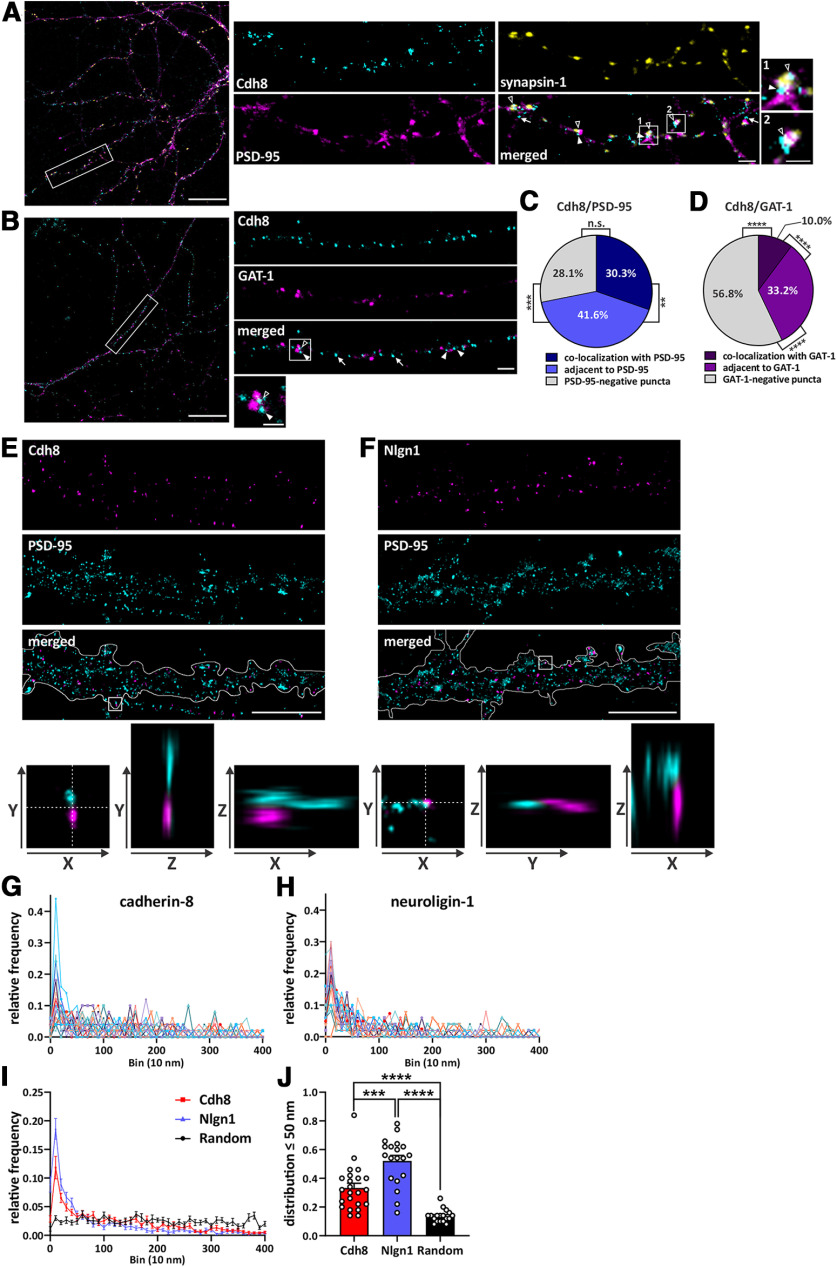
Cadherin-8 localizes to excitatory synapses. Representative confocal images showing surface expressed cadherin-8 (cyan) on dendritic segments of cultured hippocampal neurons at 15 DIV co-immunostained with either the excitatory synaptic markers (***A***) PSD-95 (magenta) and synapsin-1 (yellow), or the inhibitory synaptic marker (***B***) GAT-1 (magenta). Cadherin-8 puncta co-localizing with PSD-95-positive or GAT-1-positive puncta (open arrowheads), being adjacent to PSD-95-positive or GAT-1-positive puncta (arrowheads) and PSD-95-negative or GAT-1-negative cadherin-8 puncta (arrows) were analyzed. Scale bars: 20 μm (overview) and 2 μm (dendritic segments). Representative synaptic puncta are magnified (boxed areas; scale bar: 1 μm). The synaptic profile of cadherin-8-positive puncta were quantified and analyzed as fractions of co-localization with (***C***) PSD-95 or (***D***) GAT-1. PSD-95: **p =* *0.0019, ***p =* *0.0002, GAT-1: *p *<* *0.0001, one-way ANOVA with Tukey’s multiple comparisons test. *N* = 11–14 neurons, three independent cultures. dSTORM imaging was performed on 15 DIV hippocampal neurons immunostained for PSD-95 (cyan) and either surface-expressed (***E***) cadherin-8 (magenta) or (***F***) neuroligin-1 (magenta). Dendrites are outlined in merged images. Scale bar: 5 μm. Insets (800 nm) show 3D proximity of PSD-95 to the surface-stained proteins. Crosshair shows *yz* and *xz* planes. ***G–J***, Nearest neighbor analysis of dSTORM data. Individual surface stained (***G***) cadherin-8 and (***H***) neuroligin-1 puncta were isolated, and the nearest PSD-95 puncta was determined. ***I***, Quantification and comparison of nearest neighbor analysis for cadherin-8, neuroligin-1, and a randomized set of numbers generated between 0 and 400 nm. ***J***, Quantification and comparison of the frequency distribution of cadherin-8 and neuroligin-1 puncta and a randomized data set between 0 and 50 nm from the nearest PSD-95 puncta; ***p =* *0.0001, ****p <* *0.0001, one-way ANOVA with Tukey’s multiple comparisons test. *N* = 23 neurons (Cdh8) and 19 neurons (Nlgn1), 18 random data points, three independent cultures; 50 particles per cell were used, unless fewer particles were available.

The finding of an interaction of cadherin-8 with neuroligin-1 prompted us to further examine the subcellular localization of cadherin-8 in neurons. The specificity of the anti-cadherin-8 antibody was first validated in immunocytochemistry (Extended Data Fig. 1-1*C*). Immunofluorescence of surface-exposed cadherin-8 in 15 DIV hippocampal neurons revealed partial co-localization with the excitatory synaptic markers PSD-95 and synapsin-1 (Fig. 4*A*). The majority of cadherin-8 localized to excitatory synaptic puncta, either co-localizing with PSD-95 (30.3 ± 2.0%) or located directly adjacent to PSD-95-positive puncta (41.6 ± 2.5%), while the remainder of cadherin-8 puncta (28.1 ± 2.2%) did not localize to PSD-95-positive puncta (Fig. 4*C*). Hence, most, but not all, cadherin-8-positive puncta are associated with excitatory synapses. Conversely, the majority of the cadherin-8-positive puncta (56.8 ± 3.4%) did not associate with GAT-1-positive puncta, markers of inhibitory synapses (Fig. 4*B*,*D*). However, a small fraction of cadherin-8-positive puncta exhibited co-localization with (10.0 ± 1.8%) or were adjacent to GAT-1-positive puncta (33.2 ± 2.6%). These data suggest that cadherin-8 is enriched at/near excitatory synapses compared with inhibitory synapses, consistent with the potential role in excitatory synaptic function. To further quantify cadherin-8 presence at excitatory synapses we performed direct stochastic reconstruction microscopy (dSTORM). We examined the distance between PSD-95 and either cadherin-8 or neuroligin-1, a positive control for excitatory synaptic localization (Fig. 4*E*,*F*). For optimal two-color imaging, we used an Oxyfluor buffer system (Nahidiazar et al., 2016). 33.3 ± 3.3% of cadherin-8 puncta were located within 50 nm of a PSD-95 puncta, significantly more than a randomized set of data points (13.7 ± 1.0%; Fig. 4*G*,*I*,*J*). This recapitulates the co-localization data obtained using standard confocal microscopy. Interestingly, dSTORM imaging showed that significantly more neuroligin-1 puncta were within 50 nm of a PSD-95 punctum compared with cadherin-8 puncta and the randomized data points (52 ± 3.8%; Fig. 4*H–J*). Together, these data indicate that cadherin-8 localizes to excitatory synapses but to a lesser extent than neuroligin-1. These findings are in line with the localization of cadherin-8 and neuroligin-1 in N2a cells suggesting that these two proteins partially but not completely localize to the same cellular compartment.

### CDH8 and CDH11 levels are altered in autism-specific iPSC-derived cortical neuronal progenitor cells and organoids

**Figure 5.**
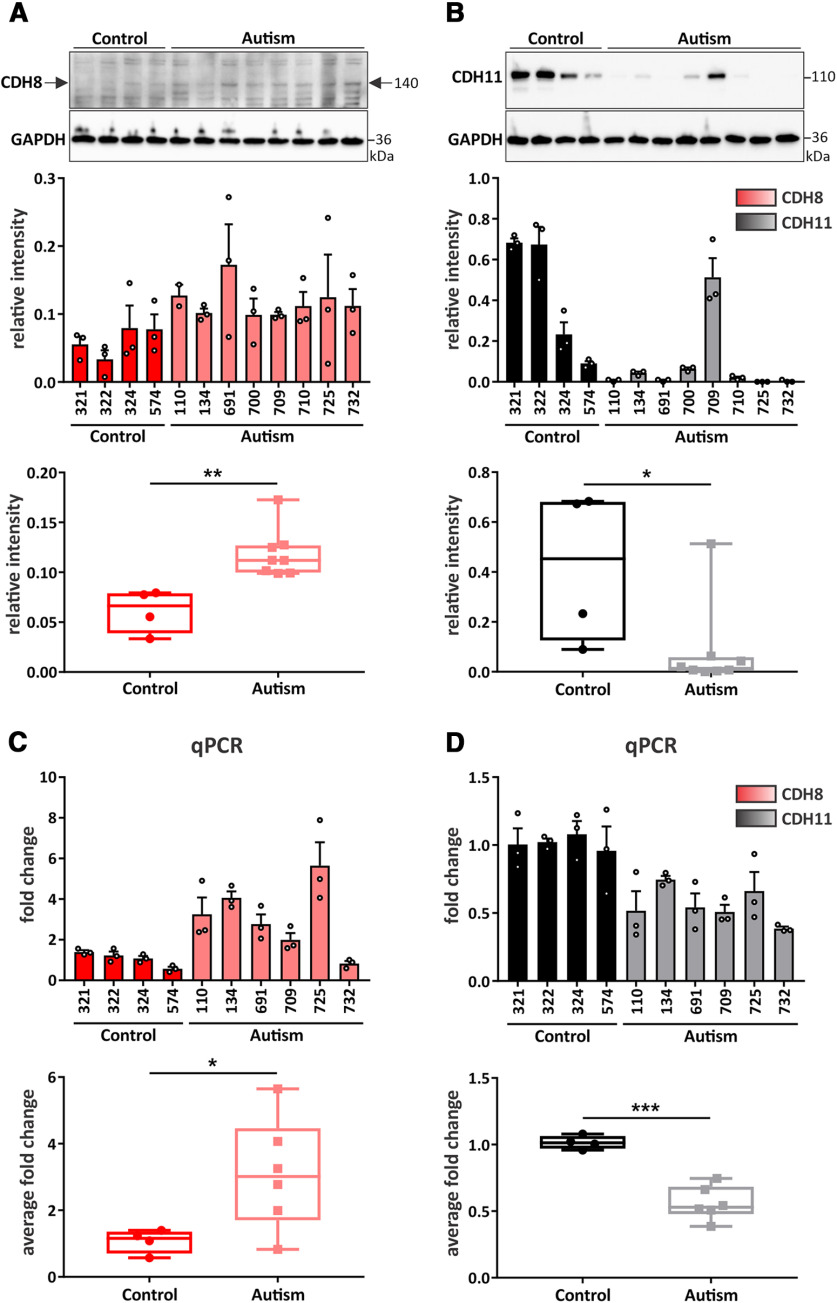
iPSC-derived cortical NPCs and organoids from autistic individuals show altered expression of CDH8 and CDH11. Western blot analysis and quantification of (***A***) CDH8 and (***B***) CDH11 expression in iPSC-derived cortical NPCs from typically-developing control and autistic individuals at 19 DIV. Quantification of the expression of each cadherin is represented as bar graph and as box and whisker plot; *p <* *0.05, **p <* *0.01; unpaired two-tailed *t* test. *N* = 4 control and 8 autistic individuals, triplicates of each individual. Cadherin signals were normalized to GAPDH. Quantification of mRNA expression of (***C***) CDH8 and (***D***) CDH11 in cortical organoids derived from iPSCs of control and autistic individuals at 60 DIV via qPCR; *p <* *0.05, ***p <* *0.001; unpaired two-tailed *t* test. *N* = 4 control and 6 autism individuals, triplicates of each individual.

CDH8 and CDH11 have been identified as autism-risk candidate genes (Hussman et al., 2011; Pagnamenta et al., 2011; Crepel et al., 2014; Cukier et al., 2014). Our findings of a dynamic expression profile of both cadherin-8 and cadherin-11 in mouse brain development and their (sub)-cellular localization in neurons and to synaptic compartments indicates that these two cadherins may function in neural circuit formation. Impaired neuronal connectivity has been suggested to represent a risk factor contributing to etiology of autism (Betancur et al., 2009; Bourgeron, 2009; Hussman et al., 2011), which prompted us to investigate the involvement of cadherin-8 and cadherin-11 in autism. As autism is a genetically complex condition with broad heterogeneity across individuals, we wanted to determine whether the levels of these cadherins are commonly altered in autism. We thus investigated the expression of CDH8 and CDH11 in human tissue using samples from autistic and typically-developing control individuals. iPSC-derived cortical neural progenitor cells (NPCs) from four neurotypical control and eight autistic individuals were cultured for 19 DIV and cell lysates were harvested for protein analysis (Table 3; Fig. 5*A*,*B*; Extended Data Fig. 1-1*A*,*B*). Although none of these individuals carried risk variants in either of these cadherins (DeRosa et al., 2018), CDH8 levels were significantly increased (control: 0.0614 ± 0.0108, autism: 0.1186 ± 0.0086; Fig. 5*A*), whereas CDH11 levels were significantly decreased (control: 0.4200 ± 0.1520, autism: 0.0821 ± 0.0621; Fig. 5*B*) in iPSC-derived cortical NPCs from autistic individuals compared with neurotypical control cells. We next performed qPCR to analyze mRNA expression of CDH8 and CDH11 in iPSC-derived cortical organoids generated from six autistic and four typically-developing control individuals that were grown in culture for 60 DIV (Table 3; Fig. 5*C*,*D*). These cortical organoids mimic early cortical development with active excitatory and inhibitory neurotransmission (Durens et al., 2020). In line with the expression profile in the NPCs, autism-specific iPSC-derived cortical organoids showed a significant increase of CDH8 (control: 1.072 ± 0.1783, autism: 3.093 ± 0.6812; Fig. 5*C*) and a concomitant decrease of CDH11 (control: 1.016 ± 0.0249, autism: 0.5603 ± 0.0517; Fig. 5*D*) compared with neurotypical control organoids. Together, these results show that both cadherin-8 and cadherin-11 levels are altered in cells derived from individuals with autism that mimic early stages of neural circuit development.

### Using *Cdh11*^−/−^ mice as a model to study potential autism-related phenotypes show increased dendrite complexity in neurons

**Figure 6.**
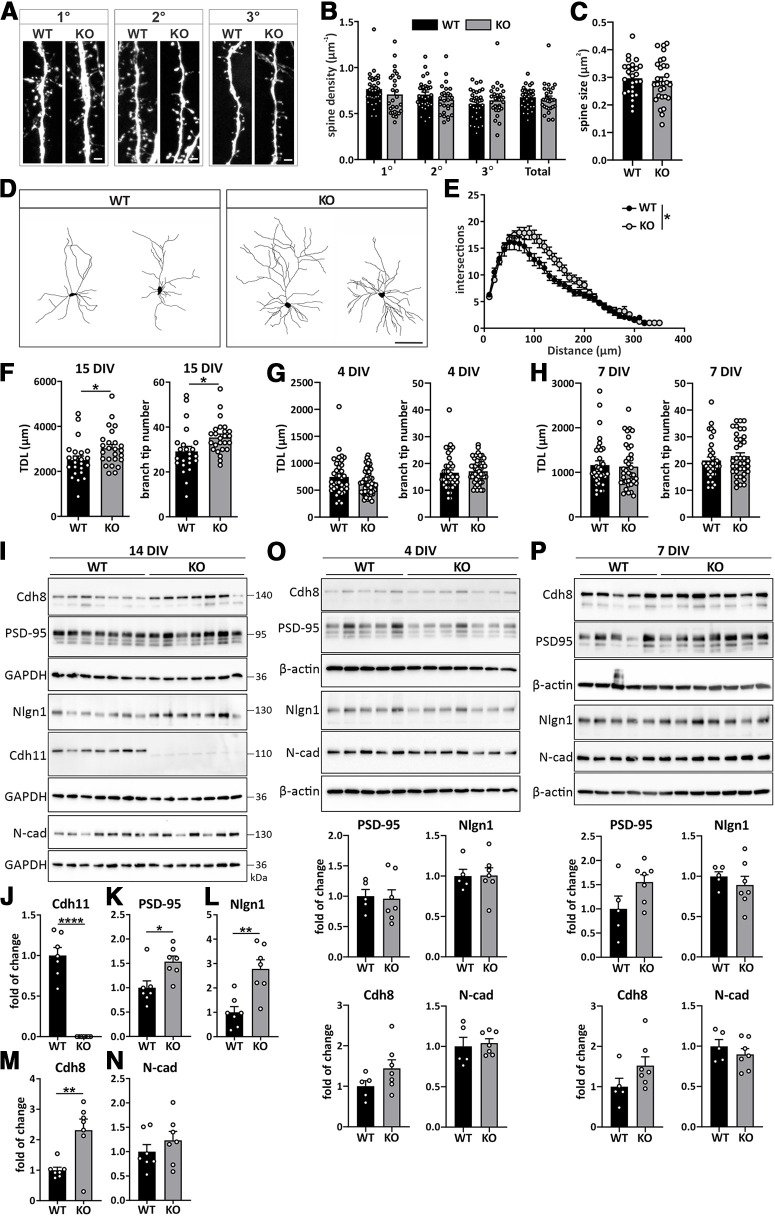
Increased dendrite complexity in *Cdh11*^−/−^ neurons is accompanied by elevated expression levels of excitatory synaptic markers. ***A***, Confocal fluorochrome images of dendritic spines from primary, secondary and tertiary dendrites from 15 DIV *Cdh11* WT and KO hippocampal neurons transfected with pLL3.7-GFP. Scale bar: 2 μm. ***B***, Quantification of the mean spine density (*N* = 33 WT and 28 KO neurons from 8 animals per genotype) and (***C***) mean spine size (26 WT neurons/7 animals and 30 KO neurons/8 animals) of 15 DIV *Cdh11* WT and KO hippocampal neurons. ***D***, Representative images of reconstructed dendritic trees from 15 DIV *Cdh11* WT and KO hippocampal neurons. Scale bar: 100 μm. ***E***, Sholl analysis of reconstructed neurons. Significant difference was determined by quantifying the area under the Sholl curve (AUC) between WT and KO neurons; *p <* *0.05, unpaired two-tailed *t* test. *N* = 23 WT and 25 KO neurons from 8 animals per genotype. Quantification of TDL and branch tip number of *Cdh11* WT and KO neurons at (***F***) 15 DIV, (***G***) 4 DIV, and (***H***) 7 DIV; *p <* *0.05, unpaired two-tailed *t* test. 15 DIV: *N* = 23 WT and 25 KO neurons from 8 animals per genotype; 4 DIV: *N* = 38 WT neurons/4 animals and 52 KO neurons/3 animals; 7 DIV: *N* = 33 WT and 37 KO neurons from 4 animals per genotype. ***I***, Western blot analysis of the expression levels of cadherin-11, PSD-95, neuroligin-1, cadherin-8, and N-cadherin in 14 DIV *Cdh11* WT and KO hippocampal cultures. Cadherin-11 is not detectable in *Cdh11* KO cultures. Signals were normalized to GAPDH. Quantification of the expression of (***J***) cadherin-11, (***K***) PSD-95, (***L***) neuroligin-1, (***M***) cadherin-8, and (***N***) N-cadherin in 14 DIV *Cdh11* WT and KO hippocampal cultures; *p < 0.05, **p < 0.01, **** *p < *0.0001, unpaired two-tailed *t* test. *N* = 7 cultures per genotype. Expression profiles and quantification of PSD-95, neuroligin-1, cadherin-8, and N-cadherin in (***O***) 4 DIV and (***P***) 7 DIV *Cdh11* WT and KO hippocampal cultures. Signals were normalized to β-actin. *N* = 5 WT and 7 KO cultures.

The *Cdh11* KO mouse has been previously shown to exhibit behaviors that are similar to autism phenotypes (Horikawa et al., 1999; Wu et al., 2021). The significant downregulation of CDH11 in autism-specific NPCs prompted us to use the *Cdh11* KO mice as a model system to examine potential pathologic phenotypes during neural circuit development and their underlying mechanisms in autism. Alterations in excitatory synapse development is one of the hallmarks of autism (Hutsler and Zhang, 2010; Forrest et al., 2018). Since we observed a peak of cadherin-11 expression during the time frame of synaptogenesis and enrichment in PSD fractions (Figs. 1*B*, 3*B*), we first examined the density of dendritic spines, the major structures that harbor excitatory synapses, in 15 DIV hippocampal cultures from *Cdh11* WT and KO mice (Fig. 6*A*). Intriguingly, we did not observe changes in the dendritic spine density on primary, secondary, and tertiary dendrites of *Cdh11*^−/−^ neurons compared with WT neurons (primary, WT: 0.7664 ± 0.0301 μm^−1^, KO: 0.7086 ± 0.0428 μm^−1^; secondary, WT: 0.7070 ± 0.0270 μm^−1^, KO: 0.6527 ± 0.0301 μm^−1^; tertiary, WT: 0.6051 ± 0.0265 μm^−1^, KO: 0.6440 ± 0.0337 μm^−1^; total, WT: 0.6778 ± 0.0194 μm^−1^, KO: 0.6607 ± 0.0299 μm^−1^; Fig. 6*B*). We also did not find significant differences in the size of dendritic spines on secondary dendrites from *Cdh11*^−/−^ neurons compared with WT neurons (WT: 0.2970 ± 0.0123 μm^2^, KO: 0.2870 ± 0.0140 μm^2^; Fig. 6*C*). We then analyzed the dendritic morphology of *Cdh11* KO and WT neurons from 15 DIV hippocampal cultures (Fig. 6*D*). Sholl analysis revealed a significant increase in the dendritic arbor complexity in *Cdh11*^−/−^ neurons compared with WT neurons (AUC, WT: 2551 ± 153.1, KO: 3051 ± 155.5; Fig. 6*E*). The increase in dendritic complexity resulted from a significant increase in TDL as well as branches as revealed by branch tip number (TDL, WT: 2509 ± 174 μm, KO: 3034 ± 162.7 μm; branch tip number, WT: 29.17 ± 2.194, KO: 35.4 ± 1.48; Fig. 6*F*). To evaluate whether the dendritic phenotype was readily present at earlier developmental time points, we further analyzed TDL and branch tip number in neurons fixed at 4 DIV and 7 DIV. In contrast to 15 DIV, TDL and branch tip number of *Cdh11*^−/−^ neurons were not significantly increased compared with WT neurons at both time points (4 DIV, TDL WT: 746.0 ± 55.14 μm, KO: 641.7 ± 31.51 μm, branch tip number WT: 16.53 ± 1.087, KO: 17.12 ± 0.600; 7 DIV, TDL WT: 1165 ± 93.83 μm, KO: 1129 ± 81.23 μm, branch tip number WT: 21.15 ± 1.312, KO: 22.76 ± 1.274; Fig. 6*G*,*H*). Together, these data indicate that loss of cadherin-11 leads to altered morphology of dendrites during a later developmental period.

### Expression levels of excitatory synaptic markers are increased in *Cdh11*^−/−^ mice

**Figure 7.**
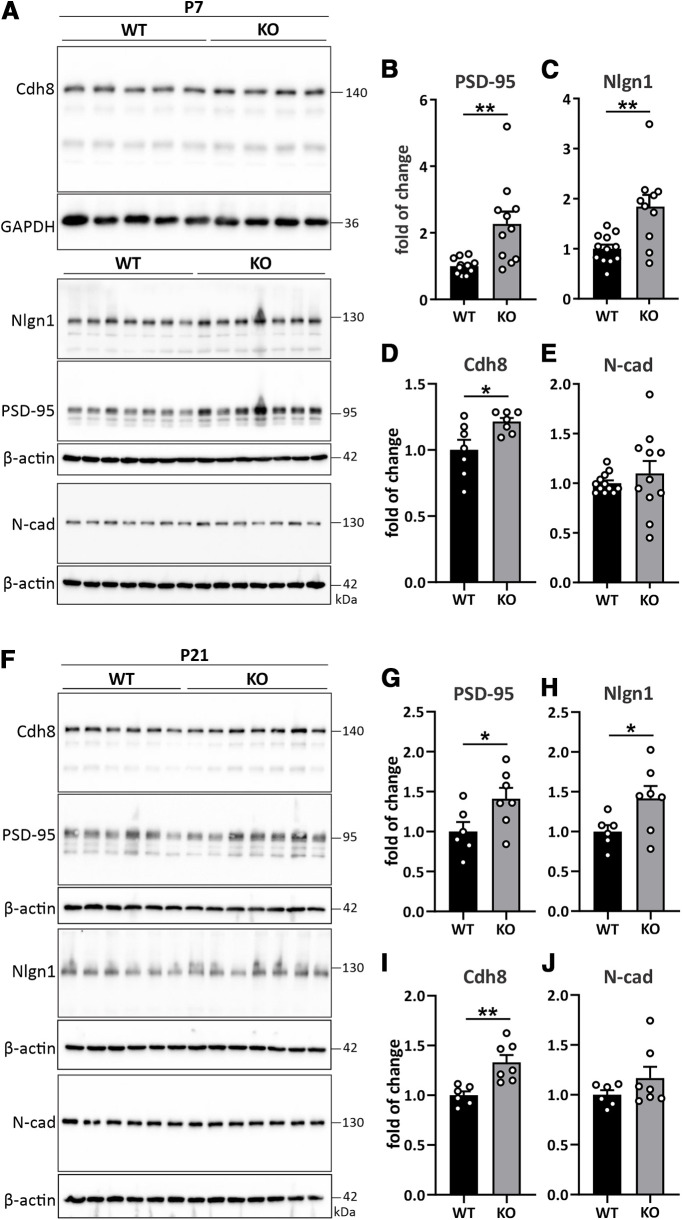
*Cdh11*^−/−^ mice show elevated expression of PSD-95, neuroligin-1, and cadherin-8 *in vivo.* ***A***, Western blot analysis of PSD-95, neuroligin-1, cadherin-8, and N-cadherin expression in whole brains of P7 *Cdh11* WT and KO mice. Cadherin-8 signal was normalized to GAPDH and Nlgn1, PSD-95, and N-cadherin signals were normalized to β-actin. One representative immunoblot for Cdh8 with five WT and four KO samples is shown. Quantification of the expression of (***B***) PSD-95, (***C***) neuroligin-1, (***D***) cadherin-8, and (***E***) N-cadherin in P7 *Cdh11* WT and KO whole brains; *p <* *0.05, **p <* *0.01, unpaired two-tailed *t* test. *N* = 7 animals per genotype (Cdh8), 12 WT and 10 KO (Nlgn1), 12 WT and 11 KO (PSD-95, N-cad). ***F***, Western blot analysis of PSD-95, neuroligin-1, cadherin-8, and N-cadherin expression in P21 forebrains of *Cdh11* WT and KO mice. Cadherin signals were normalized to β-actin. Quantification of the expression of (***G***) PSD-95, (***H***) neuroligin-1, (***I***) cadherin-8, and (***J***) N-cadherin in P21 *Cdh11* WT and KO forebrains; *p <* *0.05, **p <* *0.01, unpaired two-tailed *t* test. *N* = 6 WT and 7 KO animals.

Although synaptic density and spine size were not different in *Cdh11* KO and WT mice, the changes in dendritic complexity suggested the possibility of an overall change of synaptic structures. Therefore, we examined protein levels of the excitatory synaptic marker PSD-95 in 14 DIV hippocampal cultures prepared from *Cdh11* WT and KO mice. Western blot analysis confirmed no detectable expression of cadherin-11 in *Cdh11* KO cultures (Fig. 6*I*,*J*). However, PSD-95 levels were significantly increased in *Cdh11*^−/−^ neurons (Fig. 6*I*,*K*). We further examined protein levels of another synaptic protein, neuroligin-1, which is preferentially expressed at excitatory synapses (Song et al., 1999), and showed that neuroligin-1 was significantly increased in *Cdh11*^−/−^ cultures compared with WT cultures (Fig. 6*I*,*L*). Furthermore, we found similar expression levels of PSD-95 and neuroligin-1 in *Cdh11* KO and WT neurons at both, 4 DIV (Fig. 6*O*) and 7 DIV (Fig. 6*P*), time points that did not show any changes in dendrite morphology (Fig. 6*G*,*H*). This data suggests that the increase of dendrite complexity at a later developmental time point may likely result in an overall increase of postsynaptic sites in *Cdh11* KO neurons without changing dendritic spine density or size.

Since we identified neuroligin-1 as a selective binding partner of cadherin-8, we reasoned that cadherin-8 levels may also be altered in *Cdh11*^−/−^ neurons. Interestingly, cadherin-8 levels were not different at 4 and 7 DIV (Fig. 6*O*,*P*), but were significantly increased in *Cdh11*^−/−^ hippocampal neurons at 14 DIV (Fig. 6*I*,*M*). For all time points analyzed, there was no difference in expression levels of the classical Type I cadherin N-cadherin (Fig. 6*I*,*N–P*). We also observed this altered protein expression profile of PSD-95, neuroligin-1, and cadherin-8 in brain lysates from *Cdh11* KO and WT mice, suggesting that these changes are also detectable *in vivo* (Fig. 7). However, in contrast to the cultured neurons, the changes in protein expression levels were readily apparent at an earlier time point *in vivo* (Fig. 7*A–D*). Together, loss of cadherin-11 along with increased cadherin-8 expression in *Cdh11* KO tissue recapitulates the expression profile observed in the NPCs from autistic human subjects (Fig. 5) and further strengthens the confidence of using the *Cdh11* KO mice to investigate potential mechanistic alterations occurring in autism.

### *Cdh11*^−/−^ mice exhibit altered calcium activity and mEPSCs

**Figure 8.**
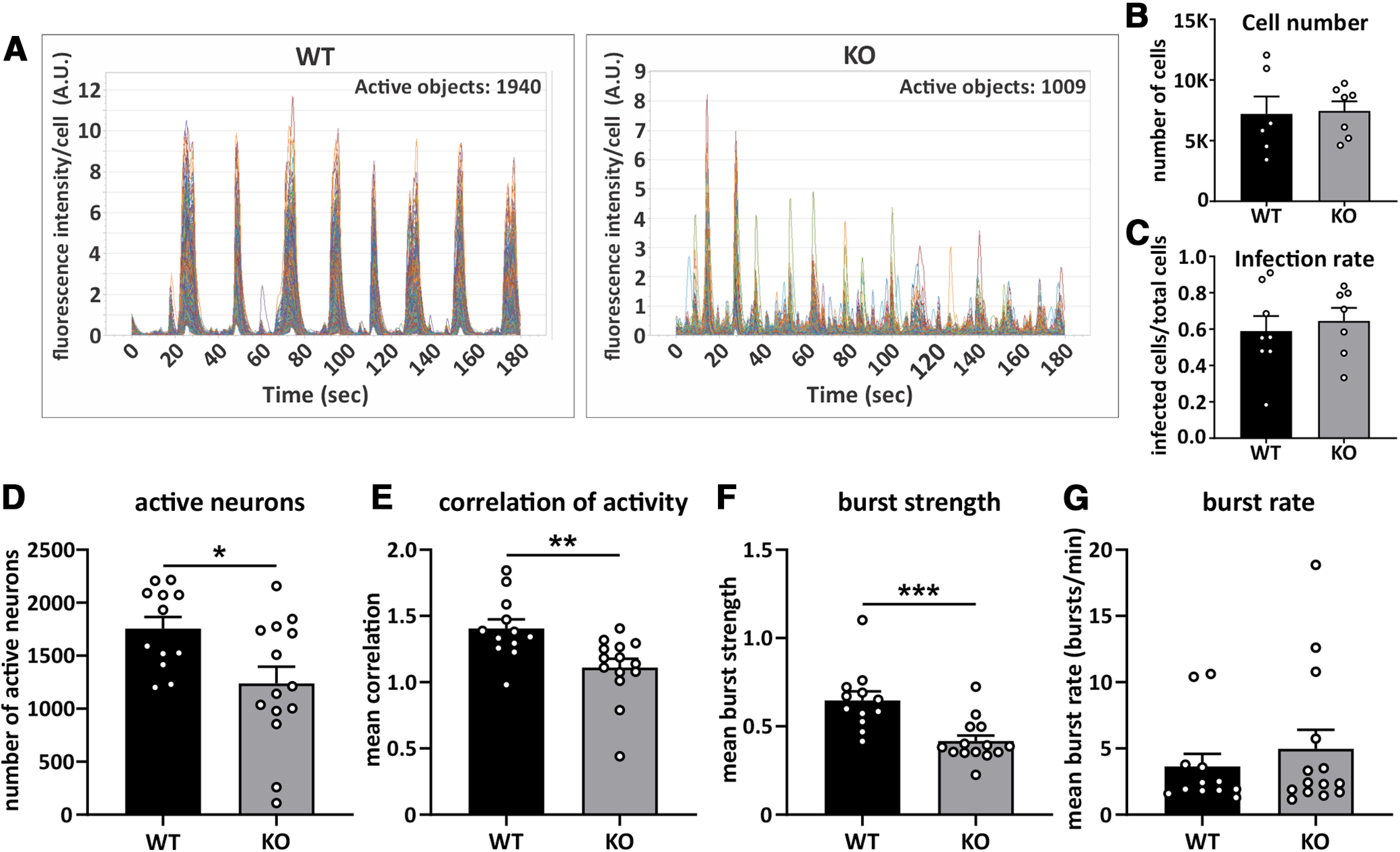
*Cdh11*^−/−^ neurons show altered calcium activity. ***A***, Example calcium current traces from *Cdh11* WT and KO hippocampal cultures at 15 DIV recorded for 3 min using NeuroBurst Orange lentivirus calcium indicator. Averaged fluorescence intensity per cell of 1940 active neurons (WT) and 1009 active neurons (KO) was plotted against time (seconds). ***B***, Total cell count of *Cdh11* WT and KO cultures using DAPI counterstain at 16 DIV. *N* = 6 WT and 7 KO cultures. ***C***, Infection rate of *Cdh11* WT and KO neurons at 16 DIV infected by the IncuCyte Neurolight Orange lentivirus. The number of infected cells was normalized to the total number of neurons counterstained with DAPI. *N* = 8 WT and 7 KO cultures. Measurement of (***D***) number of active neurons, (***E***) correlation of activity, (***F***) mean burst strength, and (***G***) mean burst rate of *Cdh11* WT and KO hippocampal neurons infected with the NeuroBurst Orange lentivirus calcium indicator recorded at 15 DIV and 16 DIV; *p <* *0.05, **p <* *0.01, ***p <* *0.001, unpaired two-tailed *t* test. *N* = 6 WT and 7 KO cultures from two different time points.

The increases of dendritic complexity and excitatory synaptic markers in *Cdh11*^−/−^ neurons prompted us to investigate whether deletion of cadherin-11 results in changes of neuronal and synaptic activity. We first transduced hippocampal neurons prepared from *Cdh11* KO and WT mice with the NeuroBurst Orange lentivirus (EssenBioscience Sartorius) and imaged network calcium activity using the IncuCyte S3 Live-Cell Analysis System for Neuroscience (EssenBioscience Sartorius) at 15 DIV (Fig. 8*A*) for 24 h. Interestingly, *Cdh11* KO cultures showed significantly fewer active neurons compared with WT cultures (WT: 1756 ± 109.9, KO: 1238 ± 159.1; Fig. 8*D*), although there was no difference in total cell number or infection rate between genotypes (cell number, WT: 7207 ± 1431, KO: 7454 ± 787.7, infection rate, WT: 0.5897 ± 0.0828, KO: 0.6452 ± 0.0719; Fig. 8*B*,*C*). In addition, the activity of *Cdh11*^−/−^ neurons was significantly less correlated when compared with WT neurons (WT: 1.405 ± 0.0681, KO: 1.111 ± 0.0657; Fig. 8*E*). *Cdh11*^−/−^ neurons further exhibited significantly reduced mean burst strengths (WT: 0.6473 ± 0.0507, KO: 0.4163 ± 0.0324; Fig. 8*F*), whereas mean burst rate was similar in *Cdh11* KO and WT cultures (WT: 3.643 ± 0.9519 min^−1^, KO: 4.983 ± 1.424 min^−1^; Fig. 8*G*). Together, these data show that *Cdh11* KO neurons exhibit a significant reduction in neuronal activity and synchrony compared with WT neurons, which suggests that synaptic input is reduced or altered.

**Figure 9.**
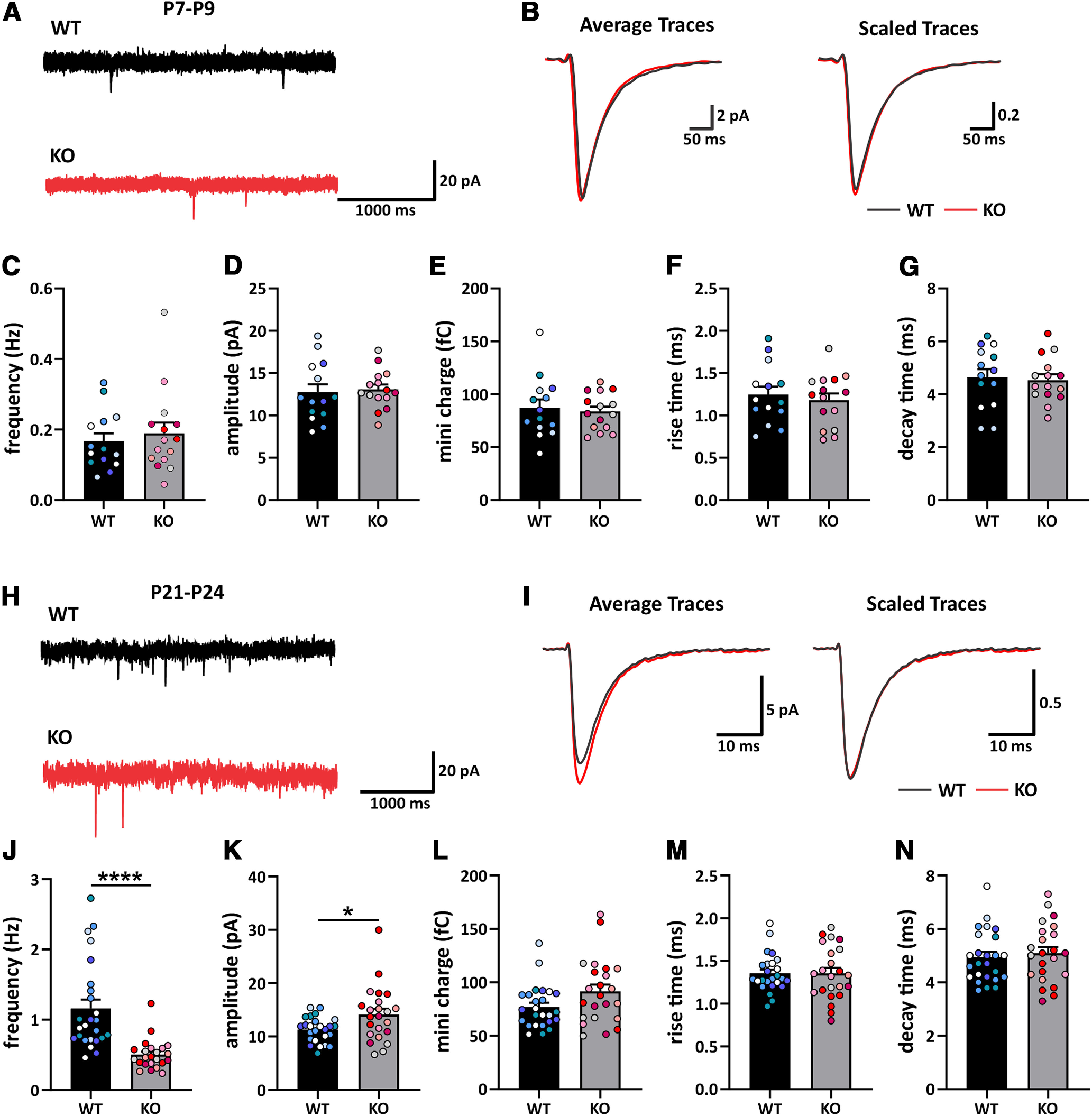
*Cdh11*^−/−^ mice exhibit changes in mEPSCs. ***A***, Example current traces recorded from whole-cell voltage-clamp of hippocampal CA1 pyramidal neurons from P7–P9 *Cdh11* WT and KO mice. ***B***, Examples of average (left) and scaled (right) mEPSC traces from P7–P9 *Cdh11* WT and KO cells. Measurement of (***C***) frequency, (***D***) amplitude, (***E***) charge, (***F***) rise time, and (***G***) decay time constant of recorded mEPSCs. *N* = 14 WT and 15 KO neurons from 5 animals per genotype. ***H***, Example current traces recorded from whole-cell voltage-clamp of hippocampal CA1 pyramidal neurons from P21–P24 mice. ***I***, Examples of average (left) and scaled (right) mEPSC traces from P21–P24 *Cdh11* WT and KO cells. Measurement of (***J***) frequency, (***K***) amplitude, (***L***) charge, (***M***) rise time, and (***N***) decay time constant of recorded mEPSCs; *p <* *0.05, ****p <* *0.0001, unpaired two-tailed *t* test. *N* = 25 WT and 23 KO neurons from 5 animals per genotype. Color codes indicate individual data points from one animal.

Because of these activity deficits, we hypothesized that excitatory synaptic input is diminished by cadherin-11 deletion. We therefore investigated hippocampal synaptic function in animals at two developmental time points. We used *ex vivo* hippocampal slices to examine the effects of *Cdh11* KO on AMPAR-mediated mEPSCs in CA1 pyramidal cells from WT and KO mice aged P7–P9 (Fig. 9*A*,*B*) and P21–P24 (Fig. 9*H*,*I*). We did not observe any differences in mEPSC frequency and amplitude in CA1 pyramidal cells from *Cdh11* KO and WT mice aged P7–P9 (frequency, WT: 0.1669 ± 0.0221 Hz, KO: 0.1891 ± 0.0308 Hz; amplitude, WT: 12.73 ± 0.9390 pA, KO: 13.07 ± 0.5904 pA; Fig. 9*C*,*D*). Also, no differences in mEPSC kinetics were observed between P7–P9 *Cdh11* KO and WT mice (charge, WT: 87.32 ± 7.684 fC, KO: 83.74 ± 4.505 fC; rise time, WT: 1.246 ± 0.0.0938 ms, KO: 1.180 ± 0.0805 ms; decay time, WT: 4.643 ± 0.3093 ms, KO: 4.533 ± 0.2207 ms; Fig. 9*E–G*). However, mEPSC frequency was significantly lower in CA1 pyramidal cells from *Cdh11* KO mice compared with WT littermates at the later time point in development, i.e., P21–P24 (WT: 1.16 ± 0.1254 Hz, KO: 0.5026 ± 0.0445 Hz; Fig. 9*J*). Conversely, mEPSC amplitude was higher in *Cdh11* KO neurons (WT: 11.28 ± 0.4684 pA, KO: 14.12 ± 1.078 pA; Fig. 9*K*). Analysis of the kinetics of CA1 mEPSCs found that there was no difference in the charge (WT: 76.92 ± 4.035 fC, KO: 91.56 ± 6.325 fC; Fig. 9*L*), rise time (WT: 1.355 ± 0.0444 ms, KO: 1.355 ± 0.0644 ms; Fig. 9*M*) or decay time constant between P21–P24 *Cdh11* KO and WT mice (WT: 4.940 ± 0.1944 ms, KO: 5.091 ± 0.2281 ms; Fig. 9*N*). The sharp reduction of mEPSC frequency in P21–P24 *Cdh11*^−/−^ CA1 neurons most likely reflects a reduction in presynaptic function or the number of functional presynaptic release sites. The concurrent increase in mEPSC amplitude is consistent with the overall increased expression of the postsynaptic markers PSD-95 and neuroligin-1. Together, these results suggest that loss of a trans-synaptic protein, such as cadherin-11, disrupts typical synaptic organization and function, and therefore neuronal activity.

Our findings from neuronal cultures and animals together indicate that loss of cadherin-11 results in altered dendritic morphology, changes in expression of excitatory synaptic markers, and disturbed synaptic transmission. These phenotypes may likely underlie neuronal alterations observed in autism.

## Discussion

In this study, we first evaluated the potential roles of the autism candidate risk genes CDH8 and CDH11 by systematically investigating their expression and localization in mouse tissue. Comprehensive analysis revealed that both of these Type II classical cadherins are expressed in the developing mouse brain at the necessary time and places to regulate crucial steps in neural circuit formation and to impact normal brain development. Further, expression analysis of CDH8 and CDH11 in NPCs and mature organoids generated from autistic individuals demonstrated that levels of the two cadherins are altered in human conditions. The analysis of the *Cdh11*^−/−^ mouse neurons further uncovered cellular and circuitry phenotypes that may be observed in autism. Our study not only describes a detailed characterization of the autism-risk genes CDH8 and CDH11, but also provides insights into novel functions of cadherin-11 in neural circuit development that may potentially be implicated in the etiology of autism.

Cadherin-8 and cadherin-11 belong to the same classical Type II cadherin subfamily. In addition to a similar structure, several studies have found similar expression profiles and functions of these two cadherins in the brain. Cadherin-8 is important for synaptic targeting in the hippocampus (Bekirov et al., 2008), striatum (Friedman et al., 2015b), and visual system (Osterhout et al., 2011; Duan et al., 2014). Cadherin-11 strongly expresses in the hippocampus and functions at glutamatergic synapses (Manabe et al., 2000; Paradis et al., 2007; Bartelt-Kirbach et al., 2010). In agreement with these studies, our spatial expression analysis shows that cadherin-8 and cadherin-11 are enriched in similar brain regions including cortex, hippocampus, and thalamus/striatum. The temporal expression analysis shows that both cadherins exhibit peak expression in postnatal brains at the time window that coincides with dendrite development and synaptogenesis, processes that have been adversely implicated in the developing autism brain (Betancur et al., 2009; Bourgeron, 2009; Hussman et al., 2011). In particular, cadherin-8 exhibited a dramatic but transient increase from embryonic stage to the first postnatal week. Protein levels quickly declined later in development and remained low in adulthood. This surge in expression indicates a putative role for cadherin-8 in regulating dendrite and synapse development, which is further supported by cadherin-8 localization to synaptic puncta. Although a direct interaction can be demonstrated between cadherin-8 and cadherin-11, we found neuroligin-1 as a selective interaction partner of cadherin-8, but not of cadherin-11, suggesting the presence of partially divergent signaling pathways and functions mediated by these two closely related cadherins. Therefore, the increase of cadherin-8 levels in the absence of cadherin-11, potentially because of compensation, may result in changes of the selective interaction between cadherin-8 and neuroligin-1 and contribute to the altered cellular and physiological phenotypes. Future studies should address a direct effect of cadherin-8, neuroligin-1, PSD95, or other proteins that may be upregulated or downregulated in the *Cdh11* KO mice to clarify their roles in the observed neuronal phenotypes.

The enrichment of cadherin-11 in SPM fractions suggests localization on both sides of the synapse, where cadherin-11 may span the synaptic cleft to act as a synaptic cell adhesion molecule. Deletion of cadherin-11 could impact the interaction between presynaptic and postsynaptic sites, which may result in disturbed synaptic function. In line with this hypothesis is the dramatic reduction in mEPSC frequency in P21–P24 *Cdh11* KO neurons, which may indicate deficits in presynaptic site number and/or function, or neurotransmitter vesicle release. The concurrent increase of mEPSC amplitude is compatible with the increased PSD-95 and neuroligin-1 protein levels and could represent a compensatory mechanism of the *Cdh11* KO neurons to reach homeostatic levels of synaptic input, or could be a direct effect of loss of cadherin-11 disrupting synaptic organization, a possibility that is consistent with the absence of an increase in dendritic spine size. The reduction in mEPSC frequency is also compatible with the observed reduction in calcium-reporter activity, as indicated by the decreased correlation of activity and lower proportion of active cells, as well as reduced mean burst strengths, reflecting an overall decrease in somatic calcium influx that could arise from diminished synaptic input. The increase in dendritic arborization may reflect structural homeostasis, where *Cdh11*^−/−^ neurons adjust the dendritic arbors and search for more synaptic input in response to reduced neuronal activity (Tripodi et al., 2008). Future studies are required to determine whether the altered morphology *in vitro* is also observed *in vivo*, where the contribution of brain area and cell type must be additionally considered.

An alternative explanation for reduced mEPSC frequency is an increased number of silent synapses. In addition to the SPM, cadherin-11 was also enriched in PSD fractions, where it may be required for anchoring or organizing receptors. Thus, depletion of cadherin-11 may impact postsynaptic AMPA-receptor expression, and consequently affect the proportion of non-functional “deaf” synapses (Voronin and Cherubini, 2004). Likewise, loss of cadherin-11, as a presynaptic adhesion molecule, could disrupt presynaptic organization or trans-synaptic connections, leading to “mute” synapses (Crawford and Mennerick, 2012). Further investigations are required to help elucidate a potential mechanism underlying the altered synaptic activity in *Cdh11* KO brains, including more detailed analysis of the presynaptic terminals, such as number, location and co-localization with PSD-95 puncta, as well as analysis of the postsynaptic site, such as number and distribution of AMPA-receptors between synapses.

Members of the cadherin superfamily have emerged as candidate risk genes for autism in multiple independent association studies (Marshall et al., 2008; Morrow et al., 2008; Depienne et al., 2009; Wang et al., 2009; Willemsen et al., 2010; Chapman et al., 2011; Hussman et al., 2011; Pagnamenta et al., 2011; Sanders et al., 2011; Camacho et al., 2012; Neale et al., 2012; O’Roak et al., 2012; Girirajan et al., 2013; van Harssel et al., 2013; Crepel et al., 2014; Cukier et al., 2014; Kenny et al., 2014). Despite these findings, no autism case reported to date has resulted from monogenic alterations in cadherins. In fact, none of the individuals with autism tested in this study harbor damaging mutations in the *CDH8* or *CDH11* genes (DeRosa et al., 2018). However, these individuals consistently show altered expression patterns in CDH8 and CDH11 throughout neural development from NPCs to more mature organoids. Despite the genetic and clinical heterogeneity seen in ASD, which is reflected by some interindividual variability shown in the expression of these cadherins in iPSC-derived NPCs, the significant differences in CDH8 and CDH11 expression suggest a potential vulnerable mechanism that involves these two cadherins. In addition to specific autism-associated mutations in cadherins, these molecules may be part of a broader pathway involving other genes mutated in autism. Indeed, some of the autism lines examined in this study also display misregulation of genes involved in cell-cell signaling and actin cytoskeleton signaling, both processes that involve cadherins (DeRosa et al., 2018). The overall decrease in synaptic activity that we observed in the *Cdh11* KO neurons is consistent with the phenotypic characterization of the iPSC-derived cortical neurons from autistic individuals performed by DeRosa et al. (2018), as they reported decreased spontaneous spiking activity, as well as decreased number of calcium transients in these neurons. The autism-specific iPSC-derived cortical neurons also exhibited decreased migration of neuronal processes (DeRosa et al., 2018). Interestingly, elevated expression of CDH11 has been observed in glioblastoma and has been associated with increased cell migration (Schulte et al., 2013). Thus, the reduced migration ability of the autism-specific neurons may stem from the decreased CDH11 expression that we observed in these autism lines. These findings further emphasize the significance of our effort to investigate the involvement of CDH11 in autism.

The variants in the CDH8 and CDH11 genes identified in individuals with autism are predicted to cause loss of function (Pagnamenta et al., 2011; Crepel et al., 2014). We found downregulation of CDH11 in autism-specific NPCs, which could mimic a loss-of-function phenotype. In contrast, NPCs from autistic individuals showed upregulation of CDH8, which may be at odds with the autism-associated loss-of-function mutations in the CDH8 gene. However, based on the known functions of cadherins, both lower and higher expression levels could directly affect neuronal connectivity by altering cell-cell adhesion, downstream signaling, and synaptic function. The autism cases investigated in this study did not carry any genetic alterations in CDH8 and CDH11 genes, and yet, they exhibited changes in protein levels of these two cadherins, further strengthening the hypothesis that cadherin signaling may represent an important pathway affected in autism. Upstream factors affecting the expression of cadherin-8 and cadherin-11 still require further investigation. Future studies should also aim to identify mechanisms that connect different cadherins to other synaptic molecules. An example of a potential connection is described here between cadherin-8 and neuroligin-1, another autism risk candidate molecule (Nakanishi et al., 2017). In conclusion, our study shows that cadherin-11 plays an important role in regulating dendritic morphology and synaptic function of excitatory neurons, and that altered expression of cadherin-11 may contribute in part to phenotypes observed in autism.

## Synthesis

Reviewing Editor: Isabel Perez-Otaño, Instituto de Neurociencias Decisions are customarily a result of the Reviewing Editor and the peer reviewers coming together and discussing their recommendations until a consensus is reached. When revisions are invited, a fact-based synthesis statement explaining their decision and outlining what is needed to prepare a revision will be listed below. The following reviewer(s) agreed to reveal their identity: Camilla Bellone. Note: If this manuscript was transferred from JNeurosci and a decision was made to accept the manuscript without peer review, a brief statement to this effect will instead be what is listed below.

The revised study addresses the role of cadherin-11 in neuronal development and maturation, with interesting phenotypes resulting from cadherin-11 loss. However, two of the reviewers considered important to include further analyses of spine morphologies and other markers of synapse maturation such as PSD95 and AMPARs. Analyzing PSD95 puncta number/size and colocalization with a presynaptic marker should help clarify the authors´ interpretation of mature but non-functional synapses.

Reviewer #1 comments:

In this new version, the authors have extensively improved the manuscript and have replied to several of the previous comments. However, there are still few questions which are not fully addressed and that could strengthens the take home message. Moreover, there are minor points that need to be clarify in the data analysis.

1. The results start with the description of figure 2, while figure 1 is only cited after figure 4 or in the method session. I suggest to move the entire figure 1 as part of figure 4 or figure 5

2. In figure 2B the y-axis show the "fold change" quantification of the WB for each time point. If this is correct, then the E14 (baseline) should be equal 1. Since this is not the case, the explanation is that the authors have plotted the relative intensity for each time point, and not the fold change normalised on E14. Authors should explain better how the analyses were performed

3. Have the authors checked the normality of the distribution of the samples? This is not well explain in the methods section.

4. For figure 3D authors should include a WB with a specific marker for glia cells, i.e. GFAP

5. The results about the effect of Cdh11 ko on synapse functionality are interesting, however some relevant experiments are missing. Authors should perform the subtype spines analysis (mushroom, stubby, thin, philopodia) in order to have a clear idea about the effect of Chd11 deletion and spine morphology. This type of analysis could help also to explain the alteration found in ephys. Regarding this, authors found that the mEPSC frequency is reduced while amplitude is increased, and they stated that the first could be explain by pre-synaptic effects while the second could be explain by the increase of PSD95 content. For the first point, authors should perform an analysis by counting the puncta between PSD95 and a presynaptic marker in Chd11 ko neurons, in this way they will have an idea about the potential synapses that can be either functional of not (that's why the spyne morphology is important). Regarding the amplitude, the fact that there is an increase of PSD95 does not necessarily mean that this contribute to the amplitude increase. Authors should check the expression of AMPARs, and this experiment is needed to better understand the role of Cdh11 in the synaptic transmission.
