# Supplementary figures and images for: Regulation of Neural Circuit Development by Cadherin-11 Provides Implications for Autism
Source: eNeuro. 2021 Jul 3;8(4):ENEURO.0066-21.2021. doi: 10.1523/ENEURO.0066-21.2021 (PMC8266214; doi:10.1523/ENEURO.0066-21.2021)

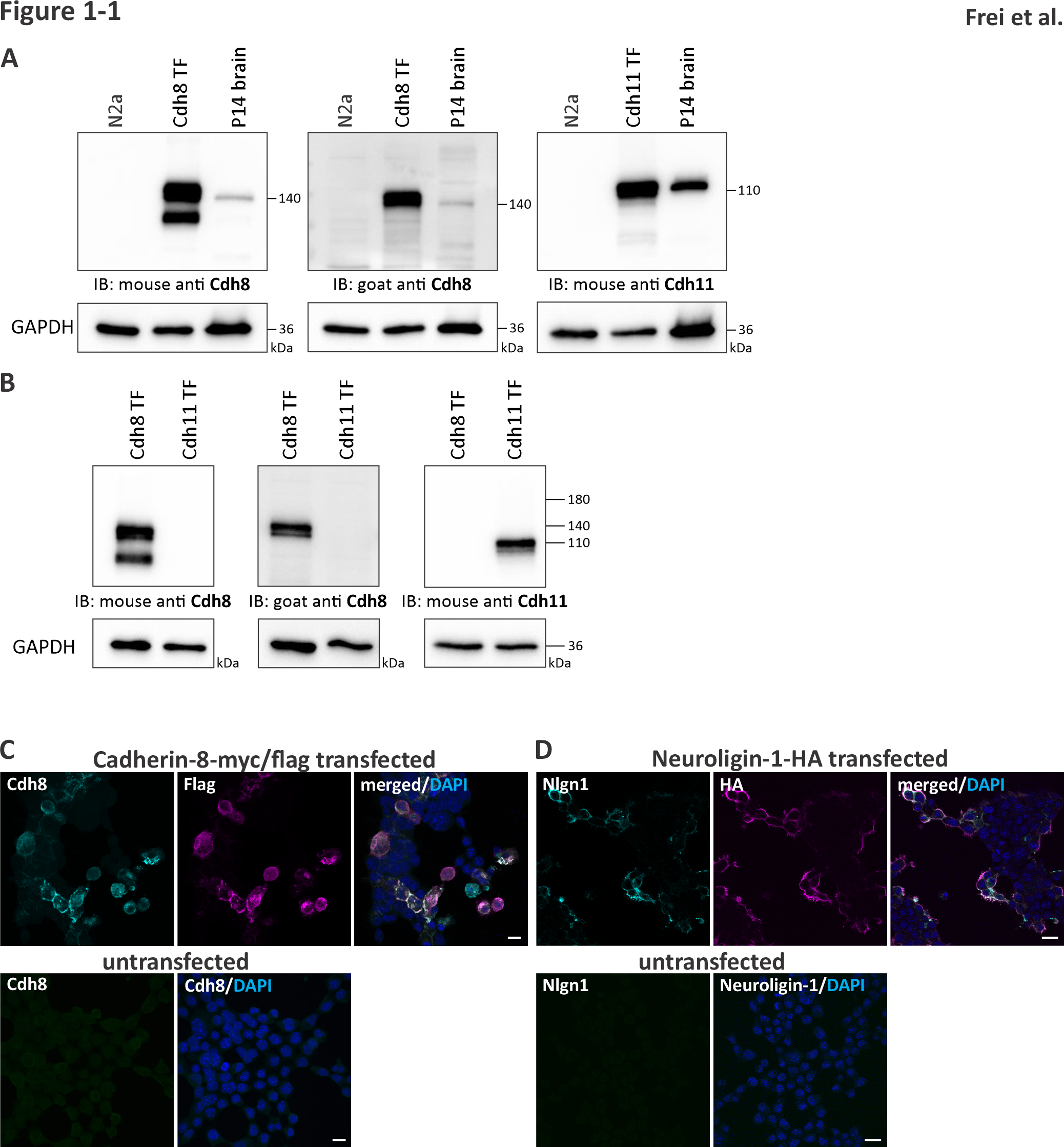

Supplement: Extended Data Figure 1-1 — Specificity of antibodies. A, Specificity of antibodies was determined by Western blotting (WB). WB of untransfected N2a cells, N2a cells transfected (TF) with myc-flag-tagged Cdh8 or flag-tagged Cdh11 and P14 mouse brain tissues were probed with mouse anti-Cdh8, goat anti-Cdh8, or mouse anti-Cdh11 antibodies. Each antibody recognizes prominent bands of predicted molecular weight [Cdh8: 140 kDa (precursor), 90 kDa (mature); Cdh11: 110 kDa], each band representing either the overexpressed protein in N2a cells or the endogenous protein in brain tissues. Note that Cdh8 and Cdh11 are not endogenously expressed in N2a cells. B, Cross-reactivity of antibodies. WB of N2a cells transfected with myc-flag-tagged Cdh8 or flag-tagged Cdh11 were probed with the same antibodies as in A. Each antibody specifically recognizes the predicted cadherin and is not cross-reacting with the other cadherin examined. GAPDH served as loading control. A, D, Specificity of antibodies tested by immunofluorescence. myc-flag-tagged Cdh8-transfected (C) or HA-tagged Nlgn-1-transfected (D) N2a cells were fixed and stained with the mouse anti-Cdh8 or mouse anti-Nlgn-1 antibody. These antibodies specifically recognize the overexpressed cadherin-8 (cyan) and its flag-tag (magenta) and the overexpressed neuroligin-1 (cyan) and its HA-tag (magenta), respectively, with characteristic localization to cell membranes and cell-cell contacts. No signals were detectable in untransfected N2a cells that were incubated with primary and secondary antibodies. DAPI signal is depicted in blue. Scale bars: 10 μm (C) and 20 μm (D). Download Figure 1-1, TIF file. [file enu-eN-NWR-0066-21-s02.tif]
